# Multistage Thermal Decomposition Kinetics of Glycidyl Azide Polymer-Based Thermoplastic Elastomers: A Constrained Deconvolution Approach

**DOI:** 10.3390/polym18050666

**Published:** 2026-03-09

**Authors:** Zhu Wang, Haoyu Yu, Shanjun Ding, Wenhao Liu, Shuai Zhao, Yunjun Luo

**Affiliations:** 1 School of Materials Science and Engineering, Beijing Institute of Technology, Beijing 100081, China; 2 AVIC Manufacturing Technology Institute, Beijing 100024, China; 3 Key Laboratory for Ministry of Education of High Energy Density Materials, Ministry of Education, Beijing Institute of Technology, Beijing 100081, China

**Keywords:** glycidyl azide polymer, thermal decomposition, asymmetric gaussian function, constrained deconvolution, activation energy, pre-exponential factor

## Abstract

Glycidyl azide polymer (GAP)-based polyurethane, a kind of energetic thermoplastic elastomer (ETPE), is a promising binder for advanced solid propellants, but its thermal decomposition involves overlapping competitive reactions that conventional single-step kinetic models cannot characterize accurately, limiting its engineering applications. To address this limitation, a constrained asymmetric Gaussian deconvolution strategy with fixed peak area ratios and shape constraints was developed in this work. This strategy was applied to resolve overlapping reaction rate curves converted from derivative thermogravimetric data of GAP-based ETPEs with 50 wt% GAP content at four heating rates of 5, 10, 15 and 20 K·min^−1^. The complex decomposition process was successfully split into five stages, assigned to azide cleavage, polyether backbone scission, carbamate cleavage, hydrocarbon product degradation and residue decomposition, with a goodness of fit of *R*^2^ > 0.998. Apparent activation energies of the five stages were determined through cross-validation by the Friedman and Flynn–Wall–Ozawa methods without prior assumption of reaction mechanisms, following the order of residue decomposition (181.4 ± 1.0 kJ·mol^−1^) > hydrocarbon product degradation (159.9 ± 1.0 kJ·mol^−1^) ≈ azide cleavage (156.5 ± 0.6 kJ·mol^−1^) > backbone scission (135.1 ± 0.7 kJ·mol^−1^) > carbamate cleavage (111.9 ± 1.1 kJ·mol^−1^). Pre-exponential factors with ln*A*_0_ values ranging from 22.2 to 34.0 were derived via the kinetic compensation effect. Finally, generalized master plots were employed to compare with classic solid-state reaction models for mechanistic insight, and the Šesták–Berggren model fit three major stages excellently (*R*^2^ > 0.996) by accounting for synergistic nucleation-growth and phase boundary mechanisms, enabling high-precision kinetic equations. It should be noted that the constrained deconvolution method proposed in this work has general applicability for kinetic analysis of GAP-based ETPEs with different formulations and other complex energetic polymer systems, while the obtained kinetic parameters are composition-specific and only applicable to the corresponding ETPE formulation studied herein.

## 1. Introduction

As a core component of advanced solid propellants, the energetic binder serves multiple critical functions, including binding particulate fillers such as oxidizers and fuels, transmitting mechanical loads [1], and contributing to energy output [2]. Its performance directly determines the propellant’s energy level and service safety. Glycidyl azide polymer (GAP)-based energetic thermoplastic elastomers (ETPEs) have emerged as preferred binders for next-generation propellants due to unique molecular structural advantages [3].

Compared with traditional inert binders such as hydroxyl-terminated polybutadiene (HTPB), the azide groups (-N_3_) on the GAP molecular chain can provide considerable energy contribution, with a theoretical specific impulse 2%~5% higher than that of HTPB [4,5,6]. Notably, GAP undergoes decomposition without the need for an external oxygen supply, which effectively optimizes the energy release efficiency of propellants. More importantly, its excellent chemical compatibility enables stable compounding with high-energy oxidizers (e.g., CL-20, RDX) and fuels (e.g., aluminum powder) [7,8,9], addressing the long-standing challenge of phase separation between traditional binders and high-energy components. Furthermore, GAP-based ETPEs integrate the processing flexibility of thermoplastic materials [10] and the mechanical durability of elastomers. They can maintain outstanding tensile strength and elongation at break within a wide temperature range of −50 °C~60 °C [11], fully meeting the operational requirements under complex working conditions.

However, the actual thermal decomposition process of GAP-based energetic thermoplastic elastomers (ETPEs) is extremely complex [12,13,14,15], involving multiple competing reactions such as azide group cleavage [16,17], urethane bond degradation [18], and main chain scission. These reactions exhibit significant overlap in their temperature ranges, and this complexity directly governs critical propellant performance metrics: storage life (e.g., stability at elevated temperatures), ignition response (e.g., decomposition gas generation rate), and combustion efficiency (e.g., energy release kinetics). Conventional single-step kinetic models are insufficient to accurately depict the thermal decomposition behavior of these materials, limiting the utility of thermal analysis data for guiding practical engineering applications. Thus, precise thermal decomposition kinetic analysis is a core prerequisite for the engineering implementation of such binders. Quantitative determination of key kinetic parameters—including apparent activation energy (*E*_a_) and pre-exponential factor (*A*_0_)—via thermogravimetric analysis (TG) and other quantitative techniques facilitates the quantitative description of the decomposition process. Accordingly, this study proposes a kinetic analysis method that combines asymmetric Gaussian functions with peak area constraints. Following the extraction of individual reaction profiles through peak deconvolution, multiple kinetic models are integrated to calculate *E*_a_ and A for each decomposition stage. This work provides indispensable theoretical support for propellant formulation optimization, safety threshold definition, storage environment design, and binder selection.

Taking GAP-based energetic thermoplastic elastomers (ETPEs) with varying hard segment contents as research objects, this study obtained thermal response curves at multiple heating rates (5, 10, 15, and 20 K·min^−1^) via TG simultaneous thermal analysis. The deconvolution of overlapping peaks and verification of peak area constraints were achieved using asymmetric Gaussian functions. Combined with the Friedman and Flynn–Wall–Ozawa (FWO) methods, the apparent *E*_a_ for each decomposition stage was determined, while the *A*_0_ of each stage was calculated by employing the kinetic compensation effect (KCE). Finally, the composite the Šesták–Berggren (S-B) model was utilized to solve the kinetic equations corresponding to each reaction stage. This study holds significant practical implications for advancing the engineering application of energetic elastomeric materials.

## 2. Experimental

### 2.1. Materials

Glycidyl azide polymer (GAP, with a number-average molecular weight of about 3700 g mol^−1^ and hydroxyl equivalent of 30.27 mgKOH/g) was supplied by Liming Research Institute of Chemical Industry (Luoyang, China) and dried under vacuum at 90 °C for 4 h before use. Dicyclohexylmethylmethane-4,4′-diisocyanate (HMDI, 98%) was obtained from Shanghai Aladdin Bio-Chem Technology Co., Ltd. (Shanghai, China). 1, 4-butanediol (BDO, 99%) was obtained from Sigma-Aldrich (St. Louis, MO, USA) and was used after vacuum drying at 60 °C for 48 h. The catalyst was obtained by dissolving dibutyltin dilaurate (DBTDL, T-12, supplied by Beijing Chemical Plant, Beijing, China) in dibutyl phthalate at a concentration of 4.8 mg/mL.

### 2.2. Preparation of the Energetic Polyurethane

Five kinds of GAP-based energetic thermoplastic polyurethanes (ETPUs) with GAP mass fractions of 10–50 wt% (at 10 wt% intervals) were synthesized via a conventional prepolymerization approach. Raw material feed amounts were determined from the hydroxyl value of GAP and targeted GAP content; dosages of HMDI and BDO were adjusted to maintain a designed NCO/OH molar ratio of 0.96 in the reaction system.

Synthetic procedures were as follows: Raw materials were weighed as calculated, and GAP was charged into a three-necked flask. After vacuum degassing at 95 °C for 1 h to remove oxygen, the flask was purged with nitrogen gas. Catalyst DBTDL and HMDI were introduced sequentially, and the mixture was stirred until the prepolymerization stage was attained. The system was then cooled to ~60 °C, followed by rapid addition of BDO and vigorous stirring for 3–5 min to ensure homogeneity.

Under a nitrogen atmosphere, the homogeneous mixture was transferred to a polytetrafluoroethylene (PTFE) mold and cured at 100 °C for a predefined duration to obtain target ETPU products. The synthesis mechanism comprises two critical steps: first, hydroxyl groups (–OH) of GAP and polyols react with HMDI to form NCO-terminated prepolymers; subsequently, these prepolymers undergo chain extension with BDO to yield high-molecular-weight polymers.

### 2.3. Thermal Decomposition Experiments

Thermogravimetric analysis (TG) was conducted using a Mettler Toledo TG analyzer (Greifensee, Switzerland; Model: TG/DSC1SF/417–2). The instrument was regularly calibrated in accordance with JJG 1135-2017 Verification Regulation of Thermogravimetric Analyzer [19], and temperature calibration was performed using high-purity indium and zinc standard reference materials prior to formal experiments to ensure the accuracy of temperature control, heating rate and mass measurement.

Prior to sample testing, a blank baseline run was performed under identical experimental conditions with an empty platinum crucible to correct for buoyancy effects during heating and eliminate systematic baseline drift. For each test, approximately 0.5–1 mg of the GAP or GAP-based ETPEs sample was placed in a platinum crucible, and heated from 30 °C to 600 °C under an argon atmosphere (40 mL·min^−1^ flow rate) at heating rates of 5, 10, 15, and 20 °C·min^−1^, respectively. All sample curves were automatically baseline-subtracted to obtain accurate online mass data, and residual mass data were collected to generate the corresponding TG curves. All experiments were repeated at least twice under identical conditions, with parallel tests showing high consistency in characteristic decomposition temperatures and mass loss trends, verifying the repeatability and reliability of the experimental results.

## 3. Methodology

### 3.1. Mathematical Background of Asymmetric Gaussian Function Deconvolution

The Gaussian function stands as one of the most widely utilized models, attributed to its notable capacity to characterize the “bell-shaped distribution” intrinsic to a wide range of natural phenomena, including spectral, chromatographic, and diffraction peaks. The core of this function lies in fitting peak signals within experimental datasets via a smooth symmetric profile, thereby enabling the extraction of critical parameters, including peak position, peak height, and full width at half maximum (FWHM). The standard mathematical form of the Gaussian function is expressed as follows:

(1) $$y=y_{0}+\frac{A}{\sigma \sqrt{2\pi }}\times \exp\left(-\frac{{\left(x-x_{c}\right)}^{2}}{2\sigma^{2}}\right)$$

where *A* is the peak area, *x*_c_ is the peak position, *σ* is the standard deviation characterizing the width of the peak, and *y*_0_ is the baseline offset.

The fundamental Gaussian function yields a peak symmetric about *x*_c_. However, experimental kinetic data from most chemical reactions rarely exhibit symmetric peaks. The asymmetric Gaussian function robustly performs peak fitting and deconvolution for such scenarios. Realized via segmentally tuning left/right decay rates, its core involves splitting *σ* into left *σ*_l_ and right *σ*_r_ standard deviations to modulate asymmetry. The asymmetric Gaussian function is expressed as the following equation:

(2) $$y=\left\{\begin{array}{c} y0+\frac{A}{\sigma_{l}\sqrt{2\pi }}\times \exp\left(-\frac{{\left(x-x_{c}\right)}^{2}}{2{\sigma_{l}}^{2}}\right),\;\;x\leq x_{c}\\ y0+\frac{A}{\sigma_{r}\sqrt{2\pi }}\times \exp\left(-\frac{{\left(x-x_{c}\right)}^{2}}{2{\sigma_{r}}^{2}}\right),\;\;x>x_{c} \end{array}\right.$$

wherein *σ*_l_ denotes the left standard deviation, governing the decay rate in the region *x* ≤ *x*_c_, while *σ*_r_ represents the right standard deviation, regulating the decay rate in the region *x* > *x*_c_. The larger the value of *σ*_l_ or *σ*_r_, the flatter the corresponding side of the peak.

A primary advantage of the asymmetric Gaussian function in peak fitting lies in the strict equivalence between its parameter *A* and the integrated area of the corresponding peak. Especially in the deconvolution of multiple overlapping peaks, this enables controlled deconvolution fitting by imposing constraints on the area ratios among individual peaks. This advantage becomes even more pronounced when peaks are severely overlapping and certain inherently existing peaks remain indistinguishable. For the subsequent deconvolution analysis, the asymmetric Gaussian function (AGF) was adopted to decompose overlapping peaks, using the numerical software OriginPro 2021 (Version 9.8.0.200).

### 3.2. Determination of Activation Energy via Model-Free Isoconversional Method

The apparent *E*_a_ is one of the most fundamental parameters in thermal decomposition kinetics, and its accuracy directly influences the outcomes of kinetic mechanism models. To minimize the error in *E*_a_, two model-free isoconversional methods—Friedman [20] and Flynn–Wall–Ozawa (FWO) [21]—were employed in this work to calculate the *E*_a_ for GAP-based ETPEs thermal decomposition. Both methods eliminate the need to assume a mechanism function, making them well-suited for multi-step complex reactions.

The kinetic process of a single thermal decomposition reaction under non-isothermal conditions generally follows the Arrhenius equation, whose expression is given below:

(3) $$\frac{d\alpha }{dT}=\frac{A_{0}}{\beta }exp\left(\frac{-E_{a}}{RT}\right)f\left(\alpha \right)$$

where *α* denotes the decomposition conversion degree, *β* (K/min) is the heating rate, *A*_0_ (min^−1^) represents the pre-exponential factor, *R* is the gas constant with a value of 8.314 J∙mol^−1^∙K^−1^, *T* (K) stands for the absolute temperature, and *f*(*α*) is the differential form of the kinetic mechanism function.

The Friedman method is a differential isoconversional approach. By rearranging the Arrhenius equation into the linear form expressed in Equation (3), this method calculates the values of ln[*β*(d*α*/d*Tα*)] and 1/*Tα* corresponding to each conversion degree *α*. Herein, an equal *α* interval of 0.05 was adopted, covering the range from 0.05 to 0.95. For a specific *α*, the *E*_a_ at this conversion degree (*E_α_*) was derived from the slope of the linear regression plot of ln[*β*(d*α*/d*Tα*)] versus 1/*Tα* using data obtained under different heating rates *β*.

(4) $$\ln{\left(\beta \frac{d\alpha }{dT_{\alpha }}\right)}_{\alpha }=l\left[A_{0}f\left(\alpha \right)\right]-\frac{E_{\alpha }}{RT_{\alpha }}$$

The Flynn–Wall–Ozawa (FWO) method, i.e., the integral isoconversional method, is based on the Arrhenius equation in its approximate integral form, as expressed in Equation (5):

(5) $$log\beta =log\left(\frac{A_{0}E_{\alpha }}{T_{\alpha }G\left(\alpha \right)}\right)-2.315-0.4567\frac{E_{\alpha }}{RT_{\alpha }}$$

wherein *G*(*α*) denotes the integral form of the mechanism function. Analogous to the aforementioned Friedman method, the *E_α_* is derived from the slope of the linear regression plot of log*β* versus 1/*T_α_* at a specific conversion degree *α*, using data acquired under different heating rates *β*.

### 3.3. Determination of the Pre-Exponential Factor via the Kinetic Compensation Effect

The pre-exponential factor *A*_0_ is a core parameter in the Arrhenius equation, whose physical essence is to characterize the effective collision frequency of reactive molecules/functional groups in the thermal decomposition reaction. Its magnitude is directly governed by the reactivity of reactive sites (e.g., azide groups, urethane bonds), molecular segment mobility, and reaction steric hindrance in the polymer system: a higher *A*_0_ indicates a greater probability of effective collisions that can trigger bond cleavage, and thus a higher tendency for reaction initiation under the same energy barrier conditions.

The kinetic compensation effect (KCE), mathematically defined by Equation (6) as the robust linear positive correlation between the apparent activation energy (E_a_) and the natural logarithm of the pre-exponential factor (lnA_0_) for a given solid-state reaction system, is a well-recognized and widely documented universal phenomenon in the thermal decomposition of polymeric materials [22,23,24]. At the molecular level, KCE originates from the synergistic compensation between the variation in the bond cleavage energy barrier of reactive functional groups and the synchronous change in the effective collision frequency of reactive species, both of which are governed by the evolution of polymer chain mobility and microphase-separated aggregated structure during continuous heating. This well-verified linear correlation provides a rigorous and reliable theoretical foundation for the accurate determination of the pre-exponential factor *A*_0_ for each decomposition stage in the present work.

(6) $$lnA_{0,x}=aE_{\alpha ,x}+b$$

wherein *a* and *b* are constants, and subscript *x* denotes the index of common kinetic function *f*_x_(*α*) in Table 1. Rearranging Equation (4) yields Equation (7):

(7) $$\ln\left[\frac{\beta \frac{d\alpha }{dT}}{f_{x}\left(\alpha \right)}\right]=-\frac{E_{\alpha ,x}}{RT_{\alpha }}+lnA_{0,x}$$

From the slope and intercept of the linear fitting plot of ln[*β*(d*α*/d*T*)/*f_x_*(*α*)] vs. 1/*T_α_*, *E_α_*_,*x*_ and *A*_0,*x*_ (*E*_a_ and *A*_0_ corresponding to the specific *f_x_*(*α*) are obtained. Substituting these values into Equation (6) gives *a* and *b*. Finally, substituting the *E_α_* obtained by the Friedman or FWO methods into Equation (6) yields *A*_0_.

### 3.4. Determination of the Kinetic Model by Means of Generalized Master Plots

It has been demonstrated that universal master plots, which are valid for experimental data acquired under arbitrary heating profiles, can be derived by introducing the generalized time *θ*, defined as follows [25,26]:

(8) $$\theta =\int_{0}^{t}\exp\left(\frac{-E_{a}}{RT}\right)dt$$

This expression can alternatively be expressed as [27]

(9) $$\frac{d\theta }{dt}=\exp\left(\frac{-E_{a}}{RT}\right)$$

that after substitution into Equation (3) yields

(10) $$\frac{d\alpha }{\alpha \theta }=A_{0}f\left(\alpha \right)$$

From Equation (10), and taking *α* = 0.5 as a reference, it follows that

(11) $$\frac{d\alpha /d\theta }{{\left(d\alpha /d\theta \right)}_{0.5}}=\frac{f\left(\alpha \right)}{f(0.5)}$$

Equation (11) reveals that for a given *α*, the reduced-generalized reaction rate (d*α*/d*θ*)/(d*α*/d*θ*)*_α_*_=0.5_ is equivalent to *f*(*α*)/*f*(0.5), provided that the correct form of *f*(*α*) is adopted.

Furthermore, the generalized reaction rate may be re-expressed as

(12) $$\frac{d\alpha }{d\theta }=\frac{d\alpha }{dt}\exp\left(\frac{E_{a}}{RT}\right)=\frac{\beta d\alpha }{dT}\exp\left(\frac{E_{a}}{RT}\right)$$

Accordingly, a quantitative relationship between the generalized reaction rate and experimental data can be established:

(13) $$\frac{d\alpha /d\theta }{{\left(d\alpha /d\theta \right)}_{0.5}}=\frac{d\alpha /dT}{{(d\alpha /dT)}_{0.5}}\frac{exp\left(E_{a}/RT\right)}{exp\left(E_{a}/RT_{0.5}\right)}$$

**Figure 1:**
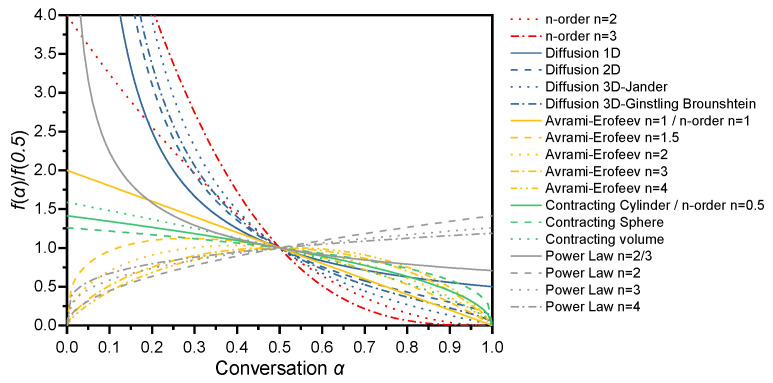
Generalized master plots corresponding to the different kinetic models in Table 1 as constructed from Equation (11). The n = 1 n-order model is mathematically identical to the Avrami-Erofeev (n = 1) model, and the n = 0.5 n-order model fully overlaps with the contracting cylinder model after normalization (constant coefficient eliminated), resulting in two pairs of coincident master curves.

The kinetic model governing the reaction can be deduced by comparing two sets of plots: the plot of reduced generalized reaction rate versus reacted fraction, calculated from experimental data via Equation (13), and the theoretical plots corresponding to different solid-state reaction models, derived from Equation (11). The master plots representing the kinetic models listed in Table 1 are presented in Figure 1.

### 3.5. Kinetic Equation Fitting Based on the Šesták–Berggren Model

Conventional single-mechanism kinetic models often fail to capture the intricacies of complex reactions, whereas the Šesták–Berggren (S-B) model [28,29,30] is a multi-parameter composite model with robust adaptability to complex reactions and employs the expression

(14) $$f\left(\alpha \right)=c\alpha^{m}{\left(1-\alpha \right)}^{n}$$

Herein, *c* denotes the proportional correction coefficient; *m* and *n* are exponents reflecting the contribution weights of the nucleation-and-growth and phase boundary reaction mechanisms, respectively, thereby enabling comprehensive characterization of these two typical reaction pathways. The model’s broad applicability across a wide conversion range ensures parameter integrity, while the well-defined physical implications of *m* and *n* facilitate mechanistic correlation. Specifically, *m* > *n* corresponds to nucleation-dominated kinetics, and *n* > *m* to phase boundary reaction dominance. These distinctive advantages establish the S-B model as the method of choice for kinetic analysis of the present system. Its differential form is given as follows:

(15) $$\frac{d\alpha }{dT}=\frac{A_{0}}{\beta }exp\left(\frac{-E_{a}}{RT}\right)c\alpha^{m}{\left(1-\alpha \right)}^{n}$$

In this work, distinct reaction stages during the thermal decomposition of GAP-based energetic polyurethane elastomers exhibit remarkably complex thermal decomposition behaviors in thermal analysis, indicating intricate decomposition reaction mechanisms. For this reason, the Šesták–Berggren (S-B) model was introduced herein; following the acquisition of *E*_a_ and *A*_0_, direct fitting of the reaction kinetic curves was performed to derive a high-precision apparent kinetic equation.

## 4. Results

### 4.1. GAP Decomposition Kinetic Curves Deconvolution via Asymmetric Gaussian Function

**Figure 2:**
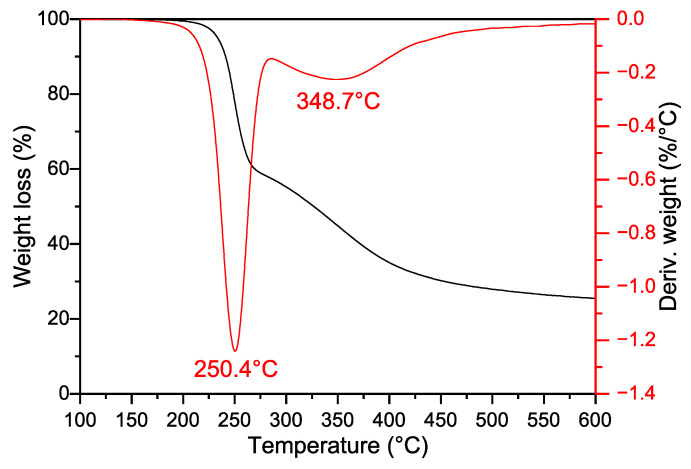
The TG and DTG curves of glycidyl azide polymer (GAP) at a heating rate of 10 °C/min.

Figure 2 presents the typical non-isothermal thermogravimetric (TG) and derivative thermogravimetric (DTG) curves of GAP during thermal decomposition. Apparently, the DTG curve consists of two overlapping peaks. As documented in numerous studies [1,2,3,4,5,6,7,8], the sharp and narrow peak with a maximum temperature of 250 °C is generated by the explosive decomposition of the -N_3_ groups, while the diffuse peak at 349 °C corresponds to the slow decomposition of the residual polyether backbone.

To facilitate kinetic calculations, the DTG curves of GAP and ETPEs were converted to the relationship between decomposition conversion rate (d*α*/d*T*) and temperature (*T*) using Equation (3):

(16) $$\alpha =\frac{w_{0}-w_{t}}{w_{0}-w_{end}}$$

where ***α*** denotes the decomposition conversion degree; ***w*_0_**, ***w*_end_**, and ***w*_t_** represent the initial sample mass, final sample mass (at the end of decomposition), and sample mass at time *t*, respectively.

**Figure 3:**
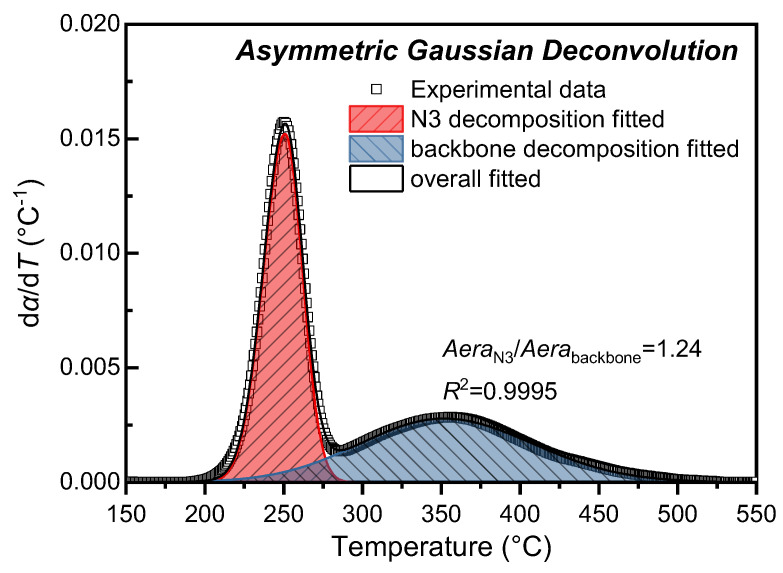
Asymmetric Gaussian deconvolution procedure for the decomposition rate curve of GAP at the heating rate of 10 °C/min. Deconvolution results for heating rates of 5, 15, 20 °C/min provided in Supplementary Materials (SM) as Figure S2.

Following the above conversion, the thermal decomposition kinetic curves of GAP at different heating rates were deconvolved using asymmetric Gaussian functions. The deconvolution result at 10 °C/min is presented as an example in Figure 3, with the remaining results provided in the Supplementary Material (SM). In DTG curves, the integrated area of each peak corresponds to the weight loss of the reaction process represented by that peak. The conversion rate curves obtained via Equation (16) exhibit the same characteristic: their deconvolved peak areas are proportional to those of the original DTG peaks, such that the ratio of peak areas remains equivalent to the ratio of weight losses from individual reactions. Benefiting from deconvolution fitting with asymmetric Gaussian functions, the area ratio between the decomposition peak of the N_3_ group and that of the GAP polyether main chain was accurately determined, with the results summarized in Table 2. The *R*^2^ between all deconvolved results and the experimental data exceeds 0.99, demonstrating that the asymmetric Gaussian function effectively achieves the deconvolution of the reaction rate curves corresponding to the two thermal decomposition stages of GAP.

As shown in Table 2, during the decomposition of GAP at different heating rates, the peak positions of both the N_3_ group and polyether backbone weight loss peaks shift progressively toward higher temperatures with increasing heating rate—an observation consistent with the dependence of thermal decomposition temperature on heating rate [31,32,33]. Moreover, the ratio of the thermal decomposition weight loss peak areas between the azide group and polyether main chain remains approximately constant at 1.24, indicating that the azide group and polyether backbone exhibit relatively stable decomposition behavior with weak dependence on heating rate.

Notably, the GAP used in this study has a number-average molecular weight (*M*_n_) of 3704 g/mol and a functionality of 2.0. Based on its repeat unit mass of 99 g/mol, the theoretical mass ratio of azide groups to the remaining main chain segments is calculated to be 0.73, which is inconsistent with the results obtained from DTG fitting. However, as observed in the TG curves (Figure 2), a residual carbide mass fraction of 25.45% remains even at 600 °C, with an average residual mass fraction of 22.51% across the four heating rates. Considering the deconvolution fitting results, the residual mass was incorporated into the molecular main chain segment when recalculating the mass ratio of azide groups to the remaining main chain. This revised calculation yielded an average ratio of 0.72, which closely approximates the theoretical value. These results confirm that the residual carbides formed after GAP thermal decomposition are predominantly derived from the polyether main chain.

### 4.2. Thermal Decomposition Behavior Analysis for GAP-Based ETPES

**Figure 4:**
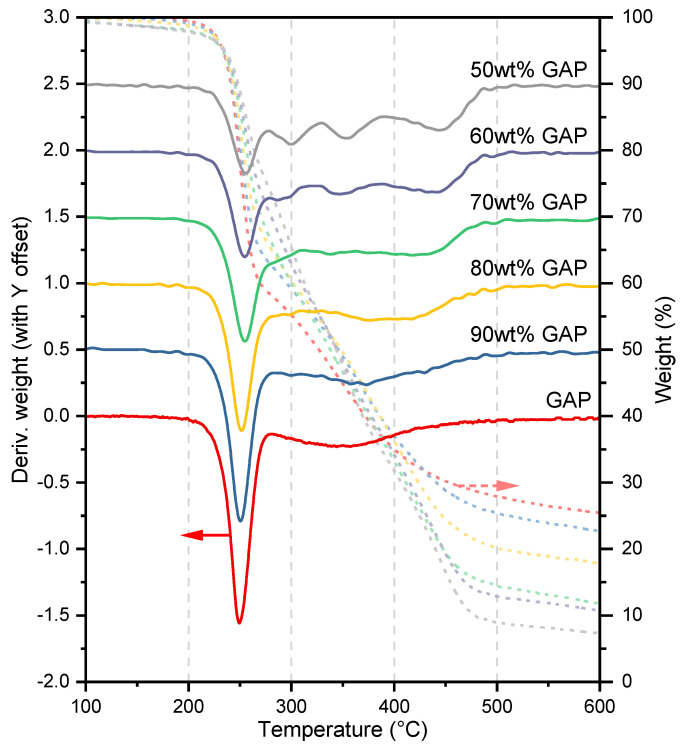
The TG and DTG curves of GAP and energetic thermoplastic elastomers (ETPEs) with different GAP contents at a heating rate of 10 °C/min. Arrows indicate the corresponding axes for the DTG (**left**) and TG (**right**) curves.

The thermal decomposition process of pristine GAP is relatively simple, with the peaks of its two independent stages being inherently symmetric. In contrast, the thermal decomposition behavior of ETPEs formed after chain extension with HMDI and BDO is more complex. As shown in Figure 4, the originally symmetric peak corresponding to polyether backbone decomposition gradually loses its symmetry as the polyurethane hard segment content increases, eventually splitting into three distinct peaks. When the content of HMDI and BDO in the resulting ETPE reaches 50 wt%, the DTG curve clearly resolves into four peaks. Therefore, subsequent kinetic studies will focus on the data obtained at this composition ratio. Notably, the completeness and resolvability of multi-step decomposition peaks in DTG curves show a strong dependence on the GAP content in ETPEs. Among the formulations with GAP content ranging from 10 wt% to 50 wt%, the DTG curve of the ETPE with 50 wt% GAP presents the most complete and well-resolved multi-step decomposition peaks, which can clearly distinguish the characteristic temperature intervals of different decomposition reactions. In contrast, with the decrease in polyurethane segment content (i.e., GAP content exceeding 80 wt%), the characteristic peaks of each decomposition reaction become indistinct and severely overlapped, with no obvious boundary between adjacent decomposition stages. This feature directly affects the accuracy of subsequent peak splitting and kinetic parameter calculation, and also defines the applicable boundary of the subsequent deconvolution strategy.

**Scheme 1:**
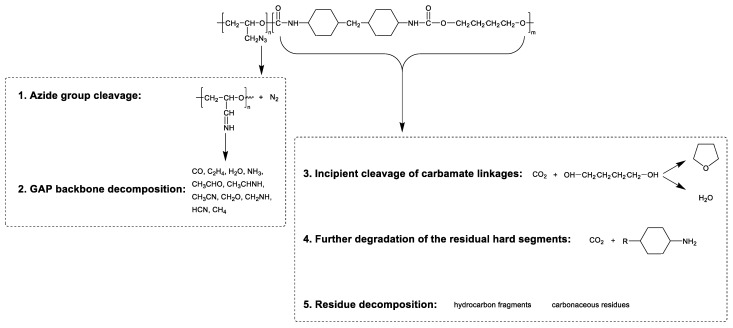
Chemical process of thermal decomposition of GAP segments [13] and polyurethane segments [34] in GAP-based ETPE.

According to the study by J.M. Cervantes-Uc et al. on the thermal decomposition of model polyurethanes synthesized from HMDI and BDO [33], which identifies three consecutive decomposition stages of the polyurethane segment (herein pre-defined as PU1 stage, PU2 stage, and polyurethane residue decomposition stage to clarify subsequent peak assignments), the decomposition peak around 285 °C (PU1 stage) is attributed to the cleavage of urethane linkages in the polyurethane segments. At this temperature, the main small-molecule products evolved are CO_2_ and the condensation product of BDO with a tetrahydrofuran structure. The peak at 325 °C (PU2 stage) corresponds to the decomposition of partial hydrocarbon products, while the peak at 445 °C represents the volatilization of amine-containing hydrocarbons generated from the further decomposition of residues. Corresponding to the DTG curve of the ETPE containing 50 wt% GAP in this study, the first sharp peak should be assigned to the decomposition of the N_3_ groups in GAP, which is consistent with the thermal decomposition behavior of pristine GAP. The temperature ranges of the second, third, and fourth peaks are fully consistent with the PU1, PU2, and polyurethane residue decomposition stages reported in J.M. Cervantes-Uc et al.’s work. However, no independent decomposition peak corresponding to the GAP polyether main chain was observed in this experiment, as its slow decomposition process rendered it most likely obscured in the spectrum. Based on the description of the thermal decomposition chemical process of HMDI-BDO polyurethane reported by J.M. Cervantes-Uc et al. [33], the inferred thermal decomposition chemical pathway of the as-prepared GAP-ETPE is illustrated in Scheme 1.

Notably, the proportion of the initial weight retained as carbonaceous residue at 600 °C in Figure 4 decreases progressively with decreasing GAP content (see Table 3 for specific values). In contrast, according to the work of J.M. Cervantes-Uc et al., polyurethanes synthesized from HMDI and BDO exhibit a residual carbon content stabilized below 2% after thermal decomposition up to 420 °C. This clearly indicates that the carbonaceous residue from the thermal decomposition of ETPEs formed by integrating GAP with the polyurethane segments is still predominantly derived from the GAP segments. Furthermore, the residual carbon generated by GAP undergoes more thorough decomposition with increasing polyurethane segment content. This also disrupts the original ratio of *A*_N3_ to *A*_backbone_ of pristine GAP. More thorough backbone decomposition enhances the proportion of *A*_backbone_. The recalculated *A*_N3_/*A*_backbone_ ratios for the ETPEs—derived by incorporating the reduced carbonaceous residue decomposition into the backbone decomposition mass while keeping the decomposition mass of N_3_ constant—are listed in Table 3.

**Figure 5:**
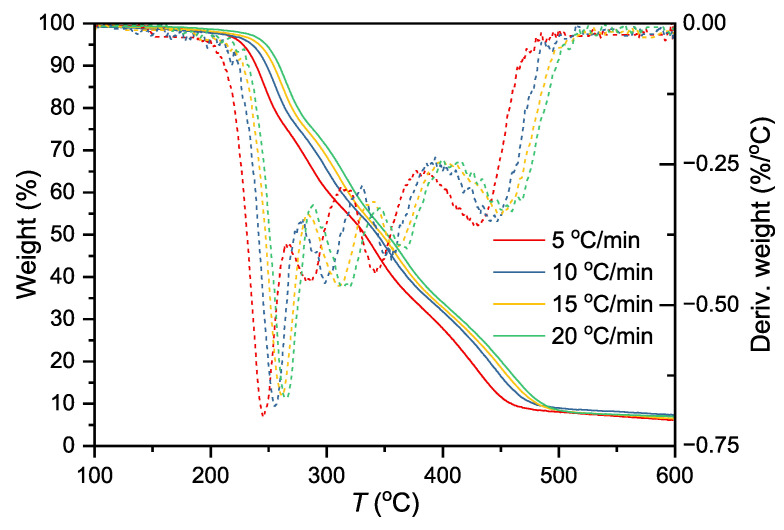
Thermal decomposition TG and DTG curves of the ETPE sample containing 50 wt% GAP at four heating rates (5, 10, 15, and 20 °C/min). Solid lines represent TG curves, and dashed lines represent DTG curves.

To accurately determine the kinetic parameters of each thermal decomposition stage for GAP-based energetic thermoplastic elastomers (ETPEs), this study designed thermal decomposition experiments with four heating rates (5, 10, 15, and 20 °C/min) using the ETPE sample containing 50 wt% GAP, and the results are presented in Figure 5. Heating rate is a critical variable regulating the thermal decomposition process in thermogravimetric (TG) analysis, and the differences in sample thermal responses induced by varying heating rates provide multi-dimensional information for kinetic interpretation: low heating rates mitigate the thermal lag effect, ensure uniform sample heating, and facilitate capturing subtle mass changes in the initial reaction stage; in contrast, high heating rates amplify differences in reaction rates via accelerated thermal accumulation, rendering the characteristic temperature boundaries of each reaction stage more distinct. Employing multiple heating rate experiments enables comprehensive coverage of the entire thermal decomposition process of GAP-based ETPEs, avoiding parameter calculation deviations caused by insufficient reaction information under a single heating rate and providing reliable data support for subsequent kinetic analysis.

Based on the obtained TG data, the Friedman and Flynn–Wall–Ozawa (FWO) methods were employed to calculate the reaction *E*_a_. The Friedman method is suitable for stage-specific analysis of multi-step reactions, while the FWO method requires no pre-assumed reaction mechanism and offers computational simplicity. Their combination enables accurate characterization and cross-validation of kinetic parameters. As indicated in the TGA curves (Figure 5), with increasing heating rate, the profiles of the four resolved peaks remain essentially unchanged, while the overall TGA and DTG curves of the sample shift toward higher temperatures. This phenomenon arises from the thermal lag effect under non-isothermal conditions, which prevents the reaction from reaching immediate equilibrium [35,36,37,38].

### 4.3. Area-Constrained Deconvolution for Multi-Stage Decomposition of GAP-Based ETPEs

To investigate the thermal decomposition kinetics of the GAP-based ETPE, the DTG curves of ETPE with 50 wt% GAP content, measured at four heating rates (5, 10, 15, and 20 °C/min), were first converted to reaction rate curves using Equation (3). Subsequently, deconvolution was performed via the asymmetric Gaussian function.

Owing to the masked decomposition peak of the polyether segment, the reaction rate curve was artificially deconvoluted into five individual peaks, designated as P_N3_, P_backbone_, P_PU1_, P_PU2_, and P_residue_, respectively. These peak notations were hereafter adopted to denote the corresponding reaction stages. A linear constraint was established via the asymmetric Gaussian function, where the area ratio of P_N3_ to P_backbone_ was strictly constrained to the value listed in Table 3. Meanwhile, the left and right widths (*σ*_1_ and *σ*_r_) of the polyether backbone decomposition peak for the ETPE were fixed, consistent with those of the GAP polyether backbone decomposition peak at the same heating rate, so as to restrict the peak shape and avoid peak distortion during the iterative process of mathematical fitting. Furthermore, from the carbonaceous residue mass in Table 3, the actual thermal weight loss of GAP segments is calculated as 0.85 times that of polyurethane segments. Accordingly, a ratio constraint was imposed in the deconvolution fitting process, defining the sum of P_N3_ and P_backbone_ areas versus that of P_PU1_, P_PU2_, and P_residue_.

**Figure 6:**
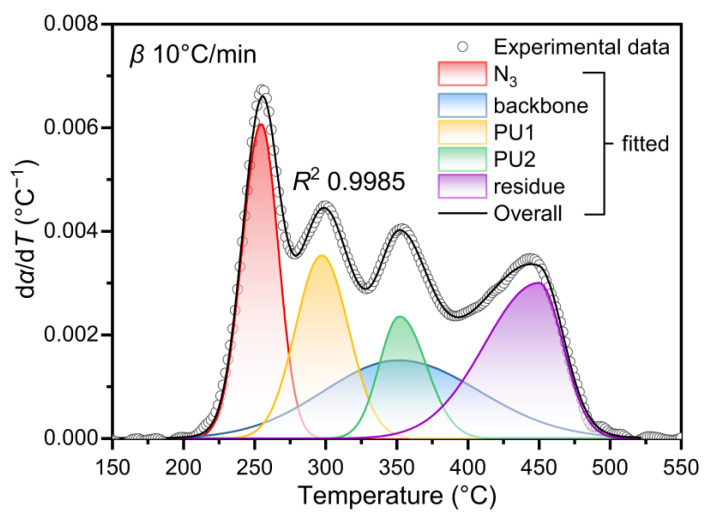
Deconvolution results of d*α*/d*T* vs. temperature curve for the GAP-based ETPE at a heating rate of 10 °C/min (Deconvolution results at 5, 15, and 20 °C/min are provided in the SM).

Figure 6 presents the deconvolution effect of the reaction kinetic curve at 10 °C/min under the above settings, while the deconvolution plots at the other three heating rates are provided in Figure S3 of the SM. The deconvolution fitting parameters of the five peaks at all heating rates are summarized in Table S1. As shown in Figure 6, the cumulative curve (solid black line) of the five asymmetric Gaussian deconvolution peaks exhibits excellent agreement with the original curve (scatter plot). Residual analysis was further performed to rigorously validate the reliability of the deconvolution strategy: the residuals were randomly distributed around the zero baseline without systematic deviation or periodic trend across all tested heating rates, and corresponding residual plots for all four heating rates are included in the SM. The *R*^2^ between the fitting results and experimental data all exceed 0.998 across the four heating rates, verifying the quantitative stability of the five-peak deconvolution strategy based on the asymmetric Gaussian function.

**Figure 7:**
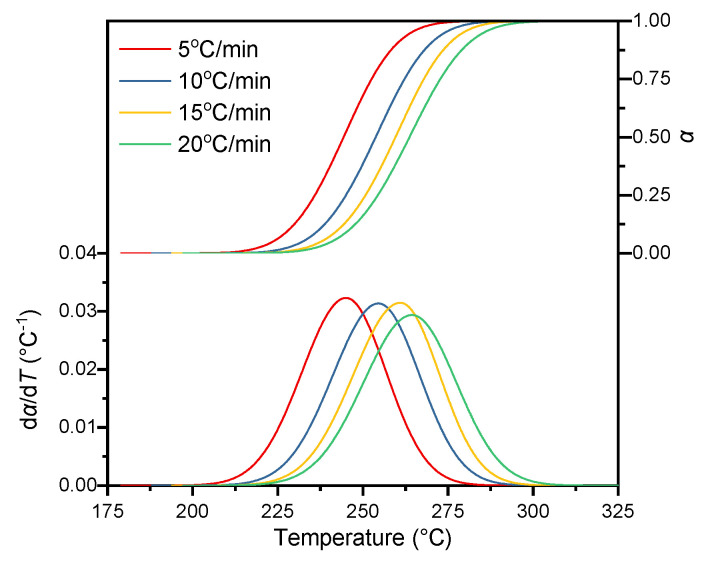
Normalized conversion rates of the N_3_ group’s thermal decomposition rate curve after asymmetric Gaussian function deconvolution; the rest of the results are given in the SM as Figure S4.

To perform kinetic analysis for each individual decomposition stage, the deconvoluted conversion rate of each stage was first normalized and then re-differentiated to obtain the curve of reaction rate versus temperature. Figure 7 presents the normalized results of the N_3_ group decomposition at the four heating rates, while those for the other reaction stages are provided in the SM. Combined with the DTG characteristic analysis of ETPEs with different GAP contents in Section 4.2, the applicability of this constrained asymmetric Gaussian deconvolution method is limited by the resolution of characteristic decomposition peaks. For ETPE systems with GAP content higher than 80 wt%, the severely overlapped decomposition peaks will lead to the risk of non-convergence in multi-peak fitting; even if convergence is achieved, it may cause serious distortion of peak position and peak shape, which will further introduce large deviations in the subsequent calculation of kinetic parameters. Therefore, the implementation of this constrained deconvolution strategy requires the DTG curve of the target system to have well-resolved characteristic peaks of each decomposition stage, so as to ensure the accuracy and reliability of the fitting results and subsequent kinetic analysis.

### 4.4. Apparent Activation Energy of Individual Decomposition Stages of GAP-Based ETPE

**Figure 8:**
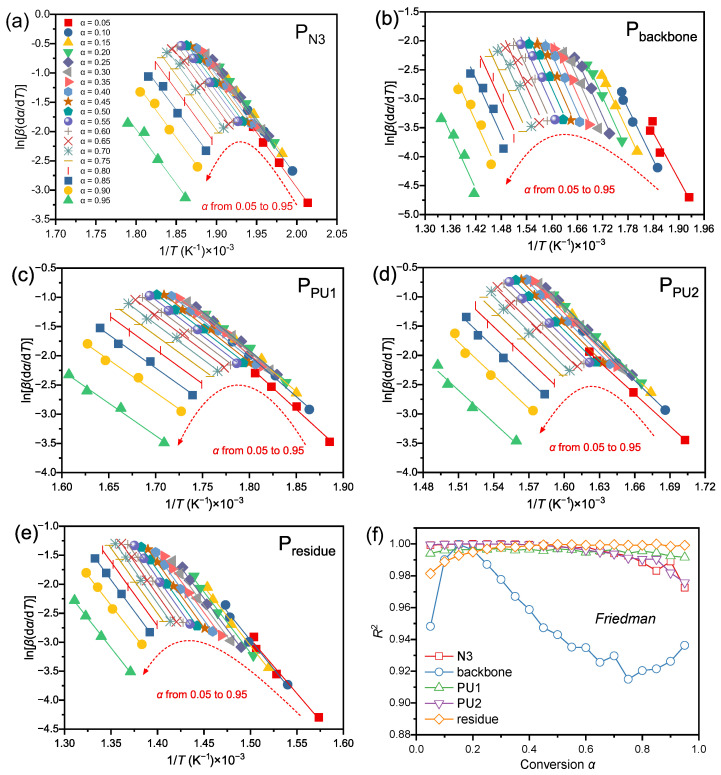
Isoconversional linear regression plots of ln[*β*(d*α*/d*T*_a_)] vs. 1/*T*_a_ for the five decomposition stages according to the Friedman method: (**a**) N_3_, (**b**) GAP backbone, (**c**) PU1, (**d**) PU2, (**e**) residue, (**f**) coefficient of determination (*R*^2^) of linear fitting for each conversion. Symbols of the same color represent the data obtained at the identical conversion level under four different heating rates (5, 10, 15, and 20 °C/min).

**Figure 9:**
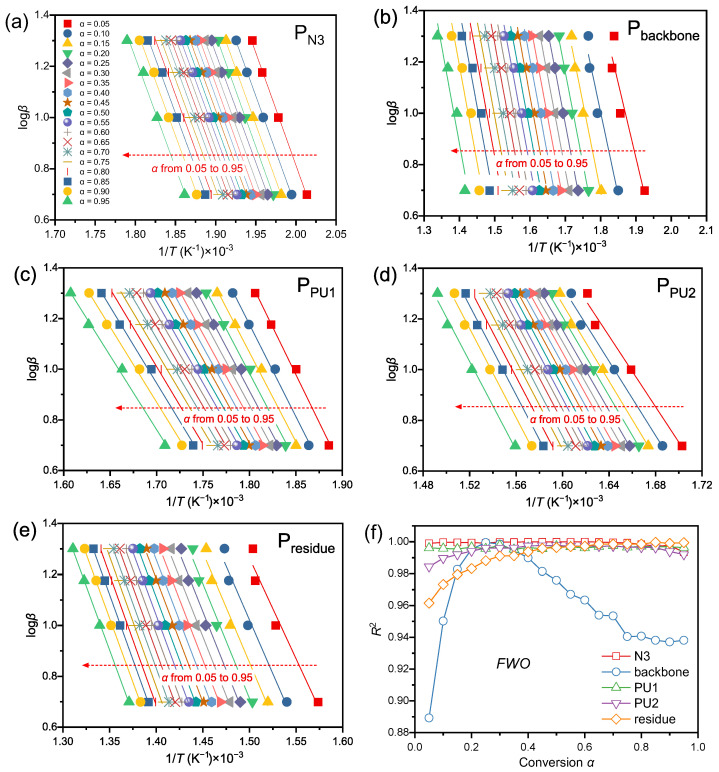
Isoconversional linear regression plots of log(*β*) vs. 1/*T*_α_ for the five decomposition stages according to the FWO method: (**a**) N_3_, (**b**) GAP backbone, (**c**) PU1, (**d**) PU2, (**e**) residue, (**f**) *R*^2^ of linear fitting for each conversion. Symbols of the same color represent the data obtained at the identical conversion level under four different heating rates (5, 10, 15, and 20 °C/min).

**Figure 10:**
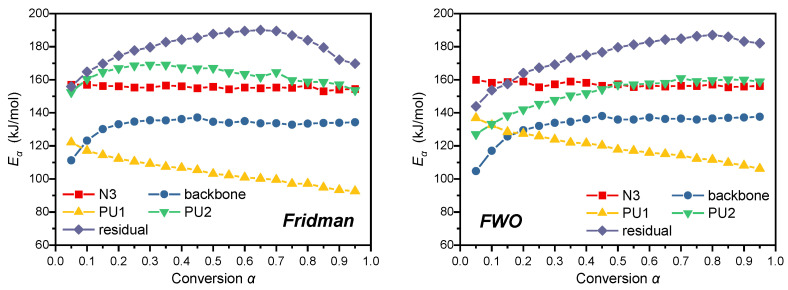
Apparent activation energies of five reaction stages as a function of conversion via the Friedman (**left**) and FWO (**right**) methods.

The apparent *E_α_* is a core parameter governing the energy barrier of thermal decomposition reactions, with its accuracy contingent upon the reliability of fitting methodologies and consistency of experimental data. In this study, two classic model-free isoconversional approaches—the Friedman differential method and Flynn–Wall–Ozawa (FWO) integral method—were adopted. Through linear fitting of the data presented in Figure 8a–e and Figure 9a–e, and their fits, yielding correlation coefficients, *R*^2^, presented in Figure 8f and Figure 9f, coupled with an analysis of the *E_α_* evolution behavior in Figure 10, the *E_α_* values corresponding to each decomposition stage were systematically determined and verified. This provides a rigorous foundation for subsequent kinetic elucidation.

For the linear fitting of ln[*β*(d*α*/d*T_α_*)] versus 1/*T_α_* using the Friedman method, all decomposition stages exhibited distinct linear correlations with a *R*^2^ exceeding 0.97 across the entire conversion range, except for the Pbackbone stage. For the Pbackbone stage, the *R*^2^ value was relatively low (0.95) at the initial reaction stage (α < 0.05), attributed to its gentle peak shape and extensive overlap with the intense peak of N_3_ decomposition, which compromised the peak deconvolution accuracy at the onset of polyether backbone decomposition. As the reaction proceeded, the *R*^2^ value first increased to above 0.99; however, when α > 0.2, it gradually decreased with increasing conversion, reaching a minimum of 0.91. This observation implies that the decomposition of the polyether backbone over a broad temperature range may involve a more complex reaction mechanism.

In the linear fitting plots of log*β* versus 1/*T_α_* using the FWO method, the fitted lines for the P_N3_ stage (Figure 9a) and P_residue_ stage (Figure 9e) presented excellent parallelism. This indicates that the kinetic nature of the reaction was consistent at different heating rates, with no rate-dependent mechanism transformation [39], which is consistent with the fitting results of the Friedman method. The fitted slopes for the P_backbone_ stage (Figure 9b) were highly consistent with those derived from the Friedman method, further verifying the stability of the *E*_a_ for polyether backbone decomposition. Cross-validation between the two methods minimized the systematic errors associated with a single approach.

Table 4 summarizes the weighted average apparent *E*_a_ at conversion degrees spanning 0.2–0.8 for the identical reaction stage, with values derived separately via the Friedman and FWO methods. This conversion range was strategically selected because the early-conversion data (α < 0.05) are inherently noisy and unreliable, which would cause unstable apparent activation energy values at low conversion, while the late-conversion stage is also subject to signal attenuation and residual side reactions that lead to unrepresentative kinetic results. Slight discrepancies exist in the conversion-dependent *E*_α_ obtained by these two approaches. The maximum relative deviation (*RD*) of 6.79% occurs in the P_PU1_ decomposition stage, followed by 3.53% in P_PU2_. In contrast, the minimum *RD* of 0.04% is observed for the P_backbone_ stage. This divergence is intrinsically linked to the mathematical underpinnings of the two methods: The Friedman method, a differential isoconversional approach relying on instantaneous reaction rate (d*α*/d*T*, i.e., DTG data), is theoretically more sensitive to reaction rate fluctuations due to the larger temperature-dependent variation amplitude of DTG data compared with integral TG data [39,40,41]. Conversely, the FWO method incorporates heating rate *β* in logarithmic form into its core equation, ensuring a rigorous linear relationship between log(*β*) and 1/*T* at constant conversion, provided the reaction kinetics (fixed *E* and *A*) remain consistent [42]. To balance calculation accuracy for rapid decomposition reactions over the 5–20 °C·min^−1^ heating rate range, the average *E*_α_ derived from both methods is adopted as the apparent *E*_a_ for subsequent kinetic investigations.

The *E*_a_ values follow the order of P_residue_ > P_PU2_ ≈ P_N3_ > P_backbone_ > P_PU1_. P_residue_ exhibits the highest *E*_a_ owing to the compact structure of its char residue, which requires higher energy to decompose. In contrast, P_PU1_ shows the lowest *E*_a_, rather than the N_3_ group decomposition, which seems counterintuitive. However, *E*_a_ is a kinetic parameter (the energy barrier to be overcome for a reaction), while bond energy is a thermodynamic parameter (the energy threshold for bond cleavage), and there is no inherent positive correlation between them. Typically, the N = N bond energy of the azido group (*ca*. 160–210 kJ·mol^−1^) [43] is much lower than that of various chemical bonds in polyurethane [44]. This aligns with the general rule that for competitive decomposition pathways of energetic materials, thermodynamic driving force (bond energy) usually outweighs kinetic barrier (*E*_a_), especially at elevated temperatures. Bonds with lower energy tend to cleave preferentially even if their cleavage requires a slightly higher *E*_a_, as they release greater overall energy [45].

### 4.5. Pre-Exponential Factor of Individual Decomposition Stages of GAP-Based ETPEs

The pre-exponential factor *A*_0_ is a pivotal kinetic parameter that characterizes the collision frequency of reactant molecules. It works together with the *E*_a_ to govern the ease of reaction initiation and directly dictates the prediction accuracy of thermal decomposition kinetic equations. In this study, the *A*_0_ values for each decomposition stage were calculated via the KCE. The core rationale for this approach lies in the linear correlation between *E*_a_ and ln*A*_0_. This characteristic has been fully validated by numerous experiments in kinetic investigations into the thermal decomposition of solid materials and thus exhibits general applicability [22,23,24].

**Figure 11:**
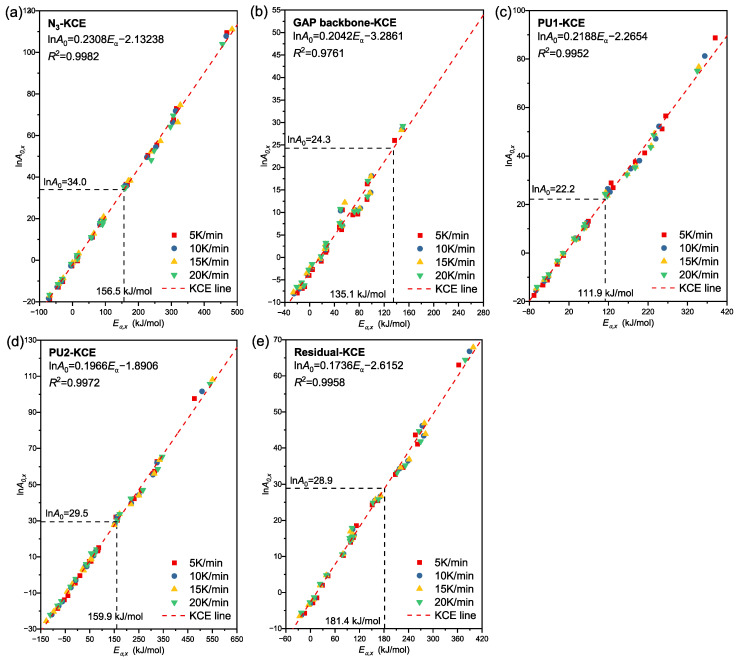
The linear KCE relationships of *E_α,x_* and ln*A*_0,*x*_ derived from 17 commonly used mechanism functions for the five reaction stages under different heating rates, along with the corresponding ln*A*_0_ obtained based on the calculated *E*_a_: (**a**) N_3_, (**b**) GAP backbone, (**c**) PU1, (**d**) PU2, (**e**) residue.

Based on the *E*_a*,x*_ and ln*A*_0,*x*_ data derived from 17 commonly used mechanism functions in Table 1 at four heating rates (all *E*_a*,x*_ and ln*A*_0,*x*_ data provided in Tables S2–S5), linear KCE fitting of ln*A*_0*,x*_ versus *E*_a*,x*_ was performed for each decomposition stage individually, with the fitting results presented in Figure 11. The results demonstrate that the *R*^2^ values of KCE linear fitting for all reaction stages except P_backbone_ are higher than 0.99, indicating that the thermal decomposition process of GAP-based ETPEs strictly conforms to the KCE within the heating rate range of 5–20 °C/min, and thus the calculation of *A*_0_ via this method is underpinned by a robust theoretical basis. Subsequently, the stage-specific *E*_a_ values obtained from the model-free method were substituted into the corresponding KCE fitted equations separately, yielding the ln*A*_0_ values of 34.0 ± 0.6, 24.3 ± 1.2, 22.2 ± 0.8, 29.5 ± 0.8 and 28.9 ± 0.8 for the P_N3_, P_backbone_, P_PU1_, P_PU2_ and P_residue_ stages, respectively.

Notably, the *A*_0_ of the P_N3_ stage is markedly higher than that of the P_backbone_ stage, which is consistent with the explosive nature of azido group decomposition—the cleavage of N = N bonds in azido groups exhibits extremely high molecular activity, and the effective collision probability is far greater than that of C-C bond cleavage in the polyether backbone [43]. Although the P_residue_ stage shows the highest *E***_a_**, its *A*_0_ is not the maximum. This is because the dense structure of char restricts molecular mobility, resulting in a smaller increase in collision frequency than the rise in energy barrier, a phenomenon that has also been reported in relevant studies on char decomposition of energetic materials [34]. The magnitude of the *A*_0_ reflects the collision efficiency of the reaction-involved groups; it synergizes with *E***_a_** to result in a distinct occurrence sequence of the five reaction stages, which differs from the sequence sorted by *E***_a_** magnitude.

### 4.6. Master Plot Comparison and Decomposition Mechanism Analysis

**Figure 12:**
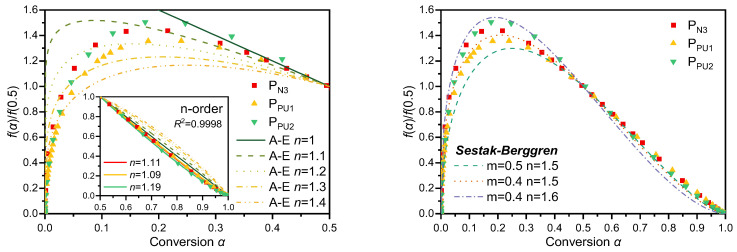
Theoretical and experimental master plot comparison for the three reaction stages of P_N3_, P_PU1_ and P_PU2_ calculated using Equation (13) (**left**); master plot of S-B model with different *m* and *n* (**right**).

**Figure 13:**
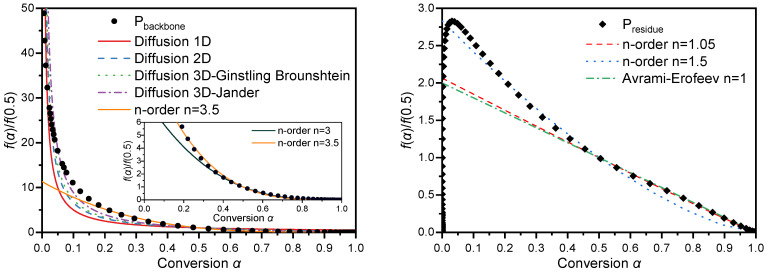
Theoretical and experimental master plot comparison for the stages of P_backbone_ (**left**) and P_residue_ (**right**).

To clarify the reaction mechanisms of each thermal decomposition stage of GAP-based ETPEs, the generalized master plot method was first employed for comparative analysis of the reaction kinetic data. Based on the generalized master plot theory established in Section 3.4, the experimentally obtained *α* and temperature data for each decomposition stage were converted into curves of reduced generalized reaction rate (d*α*/d*θ*) /(d*α*/d*θ*)_0.5_ versus *α* via Equation (13). These plots were further compared with the theoretical master plots corresponding to 17 classic solid-state reaction models listed in Table 1, with the results presented in Figure 12 and Figure 13.

Among the five peaks obtained via asymmetric Gaussian function deconvolution, the peaks of P_N3_, P_PU1_ and P_PU2_ exhibited relatively sharp and symmetric profiles, implying that these three stages may follow similar kinetic mechanisms. A comparison between master plot data of these three stages derived from Equation (13) and all theoretical master plots in Figure 1 revealed that the experimental master plots showed asymmetric parabolic profiles, a feature analogous to that of the Avrami–Erofeev model. However, as illustrated by the comparison results in Figure 12, the experimental and theoretical curves failed to achieve satisfactory agreement. In contrast, when *α* > 0.5, the decomposition behaviors of the three stages were well consistent with the *n*-order model. The exponent *n* values for the second half of the reaction process of P_N3_, P_PU1_ and P_PU2_ were determined as 1.11, 1.09 and 1.19 via fitting, with the *R*^2^ values all approaching unity. These phenomena indicate that parallel side reactions may occur in the initial stage of the three decomposition processes; as these side reactions gradually terminate in the first half of the reaction, the system transitions into a stage dominated by a single reaction mechanism. Under such circumstances, the Šesták–Berggren model demonstrated superior universality. The right panel of Figure 12 presents the master plots of the Šesták–Berggren model at different *m* and *n* values, which were found to be in close alignment with the distribution of experimental data.

**Figure 14:**
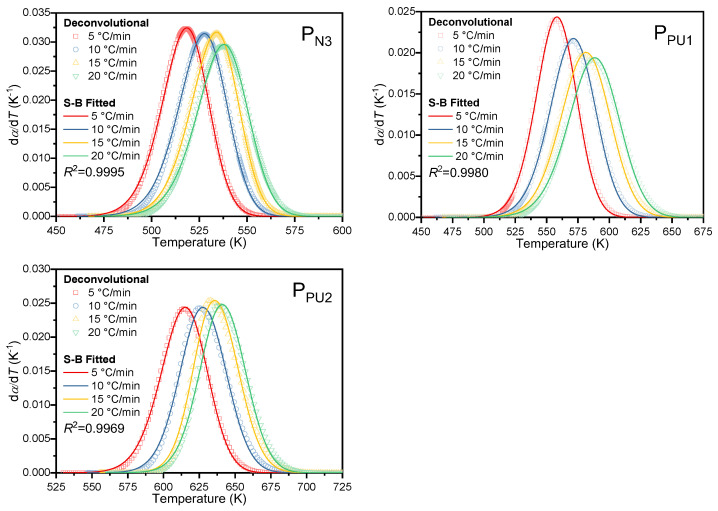
Fitting results plot of the three decomposition reaction stages of P_N3_, P_PU1_ and P_PU2_ via the Šesták–Berggren mechanism function.

Combined with the key kinetic parameters, the average *E*_a_ and *A*_0_ obtained for each stage in the preceding sections, these values were substituted into the fundamental Equation (15) for non-isothermal thermal decomposition kinetics. Subsequently, the deconvolutional data of the three decomposition stages P_N3_, P_PU1_ and P_PU2_ were fitted against Equation (15), with results shown in Figure 14. The fitted parameters *c*, *m* and *n* at different heating rates are listed in Table S6 of the SM. The fitting *R*^2^ values all exceeded 0.996, verifying the excellent applicability of the Šesták–Berggren model for describing the thermal decomposition of glycidyl azide polymer-based energetic thermoplastic elastomers.

Consistent with the definition of the S-B model in the methodology section, the exponent m reflects the contribution weight of the nucleation-growth mechanism, while the exponent n corresponds to the contribution weight of the phase boundary reaction mechanism. For the three main decomposition stages (P_N3_, P_PU1_, P_PU2_), the fitting results show that the values of both m and n are greater than 0, which directly confirms that the thermal decomposition process of these stages is jointly controlled by the nucleation-growth mechanism and the phase boundary reaction mechanism, i.e., the two mechanisms act synergistically during the reaction.

Specifically, the *n* values of all three stages are significantly higher than the corresponding m values (*n* = 1.24–1.36, *m* = 0.26–0.35), indicating that the phase boundary reaction is the dominant kinetic mechanism for these three decomposition stages, while the nucleation-growth process plays a synergistic auxiliary role. The decomposition reaction first forms nucleation sites at the active sites (such as azide groups and urethane bonds) and structural defects of the polymer matrix, which corresponds to the nucleation-growth process; subsequently, the decomposition reaction interface continuously advances from the nucleation sites and sample surface to the unreacted matrix interior, which is dominated by the phase boundary reaction mechanism. The high goodness of fit of the S-B model further verifies that the synergistic nucleation-growth and phase boundary mechanism can accurately describe the thermal decomposition kinetic process of the three main stages.

Ultimately, the kinetic equations for the three thermal decomposition stages of GAP-based ETPEs were established as follows:

(17) $${\left(\frac{d\alpha }{dT}\right)}_{N3}=\frac{5.764\times 10^{14}}{\beta }\exp\left(\frac{-156,525}{RT}\right){\times 5.056\times \alpha }^{0.26}{\left(1-\alpha \right)}^{1.24}$$

(18) $${\left(\frac{d\alpha }{dT}\right)}_{PU1}=\frac{4.460\times 10^{9}}{\beta }\exp\left(\frac{-111,885}{RT}\right){\times 2.494\times \alpha }^{0.35}{\left(1-\alpha \right)}^{1.27}$$

(19) $${\left(\frac{d\alpha }{dT}\right)}_{PU2}=\frac{6.785\times 10^{12}}{\beta }\exp\left(\frac{-159,915}{RT}\right){\times 2.400\times \alpha }^{0.35}{\left(1-\alpha \right)}^{1.36}$$

Unlike the three aforementioned reaction stages, the P_backbone_ and P_residue_ stages feature wide temperature ranges, making it difficult to ensure a consistent reaction mechanism across such broad intervals. This conclusion is further supported by the comparison between experimental and theoretical master plots presented in Figure 13. For the P_backbone_ stage, the experimental master plot exhibits a similar trend to that of the diffusion model when *α* < 0.2. As the decomposition continues, it shows close alignment with the profile corresponding to the *n*-order model with *n* = 3.5. Notably, no matching profile was identified among the master plots of all universal models for the P_residue_ stage, underscoring the considerable complexity of its reaction mechanism.

## 5. Conclusions

GAP-based ETPEs undergo highly overlapping thermal decomposition reactions that pose a major challenge to accurate kinetic characterization. This study employed an area-constrained asymmetric Gaussian function for precise deconvolution of overlapping kinetic curves, successfully resolving the complex decomposition process of ETPE with 50 wt% GAP content into five distinct stages with a goodness of fit of *R*^2^ exceeding 0.998. To ensure the reliability of kinetic parameter determination, two model-free isoconversional methods, the Friedman differential method and FWO integral method, were utilized for cross-validation to reliably quantify the apparent activation energy of each stage. Notably, all five decomposition stages strictly adhere to the KCE with a linear fitting *R*^2^ greater than 0.99, enabling robust derivation of ln*A*_0_ without prior assumption of reaction models. Furthermore, generalized master plot comparison effectively clarifies the apparent complexity of the decomposition mechanism for each stage, and the S-B model achieves excellent fitting for three relatively simple stages with *R*^2^ > 0.996, yielding high-precision kinetic equations that account for synergistic nucleation-growth and phase boundary mechanisms.

The constrained asymmetric Gaussian deconvolution strategy established in this work provides a rigorous and practical analytical protocol for kinetic analysis of GAP-based ETPEs with different formulations and other complex energetic polymer systems with multi-step overlapping decomposition reactions. It should be emphasized that the kinetic parameters obtained in this work are composition-specific, which are only applicable to the 50 wt% GAP content ETPE formulation studied herein, and cannot be directly generalized to other GAP-based ETPE formulations. Meanwhile, the applicability of this deconvolution method is limited by the resolution of characteristic decomposition peaks: for ETPE systems with GAP content higher than 80 wt%, severely overlapped decomposition peaks will lead to the risk of non-convergence or serious peak distortion in multi-peak fitting, which should be paid special attention to in subsequent applications.

Collectively, this work provides critical theoretical support for the formulation optimization, thermal safety assessment and engineering application of GAP-based ETPEs in advanced solid propellants.

## Figures and Tables

**Table 1:** Commonly used theoretical kinetic models.

Model No.	*f*_x_(*α*)	Description	
1	1 − *α*	*n*-order Models (1 − *α*)*^n^*	*n* = 1
2	(1 − *α*)^2^		*n* = 2
3	(1 − *α*)^3^		*n* = 3
4	1/2*α*	Diffusion Models	1-D
5	[−ln(1 − *α*)]^−1^		2-D
6	[(3/2)(1 − *α*)^2/3^]/[1 − (1 − *α*)^1/3^]		3-D Jander *n* = 2
7	[(3/2)(1 − *α*)^1/3^]/[1 − (1−*α*)^1/3^]		Ginstling-Brounshtein
8	2(1 − *α*)[−ln(1 − *α*)]^1/2^	Avrami–Erofeev (A-E) Models n ≠ 1 *n*(1 − *α*)[−ln(1 − *α*)]^1−1/*n*^	*n* = 2
9	3(1 − *α*)[−ln(1 − *α*)]^2/3^		*n* = 3
10	1.5(1 − *α*)[−ln(1 − *α*)]^1/3^		*n* = 1.5
11	4(1 − *α*)[−ln(1 − *α*)]^3/4^		*n* = 4
12	2(1 − *α*)^1/2^	Geometric Contraction Models	Contracting Cylinder
13	3(1 − *α*)^1/3^		Contracting Sphere
14	(2/3)*α*^−1/2^	Power Law Models *nα*^1/^*^n^*	*n* = 2/3
15	2*α*^1/2^		*n* = 2
16	3*α*^1/3^		*n* = 3
17	4*α*^1/4^		*n* = 4

**Table 2:** The deconvolution results of the decomposition rate curve of the N_3_ group and GAP polyether backbone obtained via asymmetric Gaussian functions at different heating rates.

β (°C/min)	Peak Temperature (°C)		Deconvoluted Peak Area Ratio *A*_N3_/*A*_backbone_	*R* ^2^
	N_3_	Backbone		
5	242.0	339.2	1.24	0.9990
10	250.4	348.7	1.24	0.9995
15	259.9	367.0	1.25	0.9991
20	264.3	391.3	1.24	0.9981

**Table 3:** The carbonaceous residue content at 600 °C for the ETPEs with different GAP contents.

GAP Content (wt%)	Carbonaceous Residue Content at 600 °C (wt%)	Recalculated *A*_N3_/*A*_backbone_
100	25.46	1.24
90	22.68	1.23
80	17.8	1.13
70	11.82	0.99
60	10.75	1.01
50	7.29	0.94

**Table 4:** Activation energies of four thermal decomposition stages for the GAP-based ETPE calculated via the Friedman and FWO methods.

	Weighted Average *E_a_* and Confidence Intervals/kJ·mol^−1^				
	P_N3_	P_backbone_	P_PU1_	P_PU2_	P_residue_
Friedman	155.8 ± 0.7	135.1 ± 1.2	104.3 ± 1.1	165.6 ± 0.9	184.9 ± 0.9
FWO	157.2 ± 0.4	135.2 ± 1.2	119.5 ± 1.1	154.3 ± 1.1	177.9 ± 1.1
Average	156.5 ± 0.6	135.1 ± 0.7	111.9 ± 1.1	159.9 ± 1.0	181.4 ± 1.0
*RD*	0.45%	0.04%	6.79%	3.53%	1.93%

## Data Availability

The original contributions presented in this study are included in the article/Supplementary Material. Further inquiries can be directed to the corresponding authors.

## Supplementary Materials

The following supporting information can be downloaded at https://www.mdpi.com/article/10.3390/polym18050666/s1: Figure S1. The TG and DTG curves of GAP at different heating rates. Figure S2. Asymmetric Gaussian deconvolution procedure for the decomposition rate curve of GAP at the heating rates of 5, 15, 20 °C /min. Figure S3. Deconvolution results of d*α*/d*T* vs. temperature curve for the ETPE with 50 wt% of GAP content at the heating rates of 5, 15 and 20 °C/min. Figure S4. Normalized conversion rate curve of the deconvolution peaks of GAP-based ETPE: (a) P_backbone_, (b) P_PU1_, (c) P_PU2_, (d) P_residue_. Figure S5. Residual distribution of deconvolution fitting for the dα/dT curve of ETPE with 50 wt% GAP content at a heating rate of 5 °C/min. Figure S6. Residual distribution of deconvolution fitting for the dα/dT curve of ETPE with 50 wt% GAP content at a heating rate of 10 °C/min. Figure S7. Residual distribution of deconvolution fitting for the dα/dT curve of ETPE with 50 wt% GAP content at a heating rate of 15 °C/min. Figure S8. Residual distribution of deconvolution fitting for the dα/dT curve of ETPE with 50 wt% GAP content at a heating rate of 20 °C/min. Table S1. Deconvolution fitting parameters of the five thermal decomposition stages at different heating rates. Table S2. *E_α,x_* and ln*A*_0,*x*_ values of GAP-based ETPE thermal decomposition at 5 K/min derived from 17 universal kinetic mechanism functions. Table S3. *E_α,x_* and ln*A*_0,*x*_ values of GAP-based ETPE thermal decomposition at 10 K/min derived from 17 universal kinetic mechanism functions. Table S4. *E_α,x_* and ln*A*_0,*x*_ values of GAP-based ETPE thermal decomposition at 15 K/min derived from 17 universal kinetic mechanism functions. Table S5. *E_α,x_* and ln*A*_0,*x*_ values of GAP-based ETPE thermal decomposition at 20 K/min derived from 17 universal kinetic mechanism functions. Table S6. The parameter fitting results of the S-B model for the deconvolution peaks corresponding to the P_N3_, P_PU1_ and P_PU2_ stages.
