# Supplementary material for: Multistage Thermal Decomposition Kinetics of Glycidyl Azide Polymer-Based Thermoplastic Elastomers: A Constrained Deconvolution Approach
Source: Polymers (Basel). 2026 Mar 9;18(5):666. doi: 10.3390/polym18050666 (PMC12986969; doi:10.3390/polym18050666)
Supplement: Supplementary file 1 [file polymers-18-00666-s001.zip › polymers-4172666-supplementary.pdf]

Multistage Thermal Decomposition Kinetics of Glycidyl Azide Polymer - Based Thermoplastic Elastomers: A Constrained Deconvolution Approach

Supplementary Material

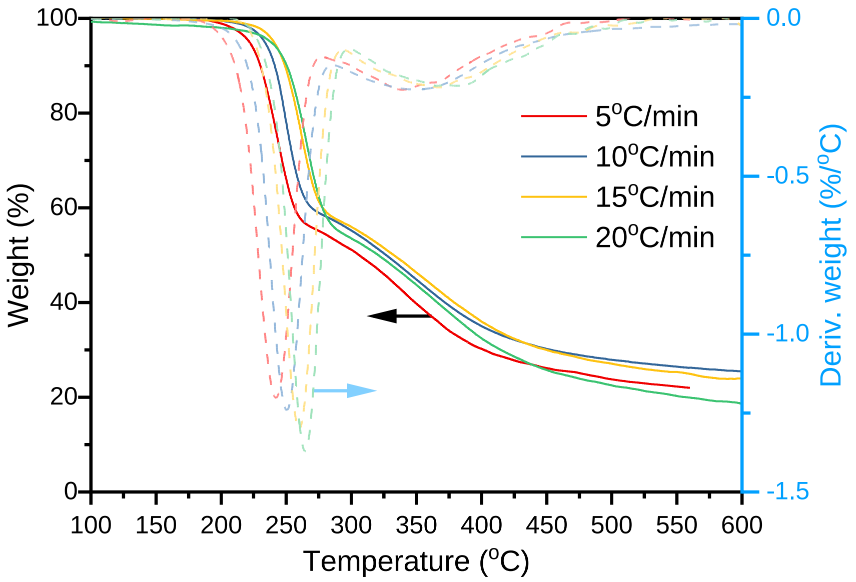

Figure S1 The TG and DTG curves of GAP at different heating rates.

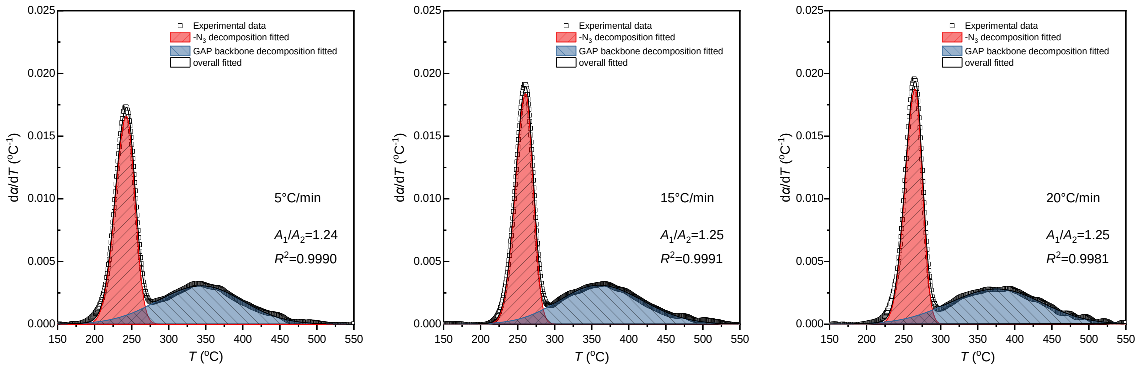

Figure S2 Asymmetric Gaussian deconvolution procedure for the decomposition rate curve of GAP at the heating rate of 5, 15, 20 °C /min.

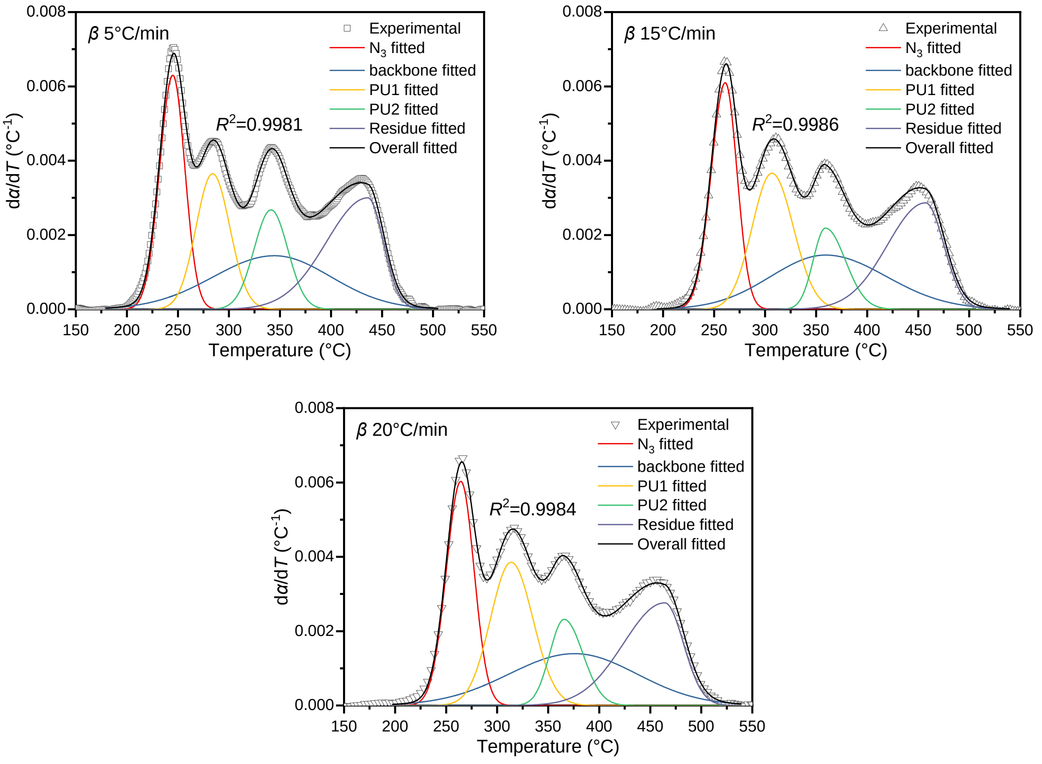

Figure S3 Deconvolution results of dα/dT *vs*. temperature curve for the ETPE with 50wt% of GAP content at the heating rate of 5, 15 and 20 °C/min.

Table S1 Deconvolution fitting parameters of the five thermal decomposition stages at different heating rate.

**β °C/min**	**Peaks**	**Fitting parameters**				**R^2**
		**A**	**x_c**	**σ_l**	**σ_r**	
**5**	**P_{N3}**	0.1949	245.0	18.27	16.66	0.9981
	**$P_{backbone}$**	0.2083	344.6	84.70	78.30	
	**P_{PU1}**	0.1517	284.0	23.02	23.87	
	**P_{PU2}**	0.1107	341.2	23.46	23.21	
	**$P_{residue}$**	0.2097	436.1	57.25	21.59	
**10**	**P_{N3}**	0.1935	254.6	18.95	17.04	0.9985
	**$P_{backbone}$**	0.2067	351.2	74.55	80.18	
	**P_{PU1}**	0.1646	297.1	25.87	26.65	
	**P_{PU2}**	0.0969	351.9	20.15	26.32	
	**$P_{residue}$**	0.2071	449.9	53.92	23.87	
**15**	**P_{N3}**	0.1937	261.0	19.55	16.29	0.9986
	**$P_{backbone}$**	0.2069	359.2	75.76	84.19	
	**P_{PU1}**	0.1848	306.7	27.31	29.60	
	**P_{PU2}**	0.0860	359.1	16.87	27.54	
	**$P_{residue}$**	0.1982	457.1	52.58	25.46	
**20**	**P_{N3}**	0.2053	264.4	20.06	18.36	0.9984

**$P_{backbone}$**	0.2194	376.0	91.03	86.52
**P_{PU1}**	0.2007	313.7	28.67	30.10
**P_{PU2}**	0.0938	365.6	19.81	25.88
**$P_{residue}$**	0.2027	464.2	57.00	25.84

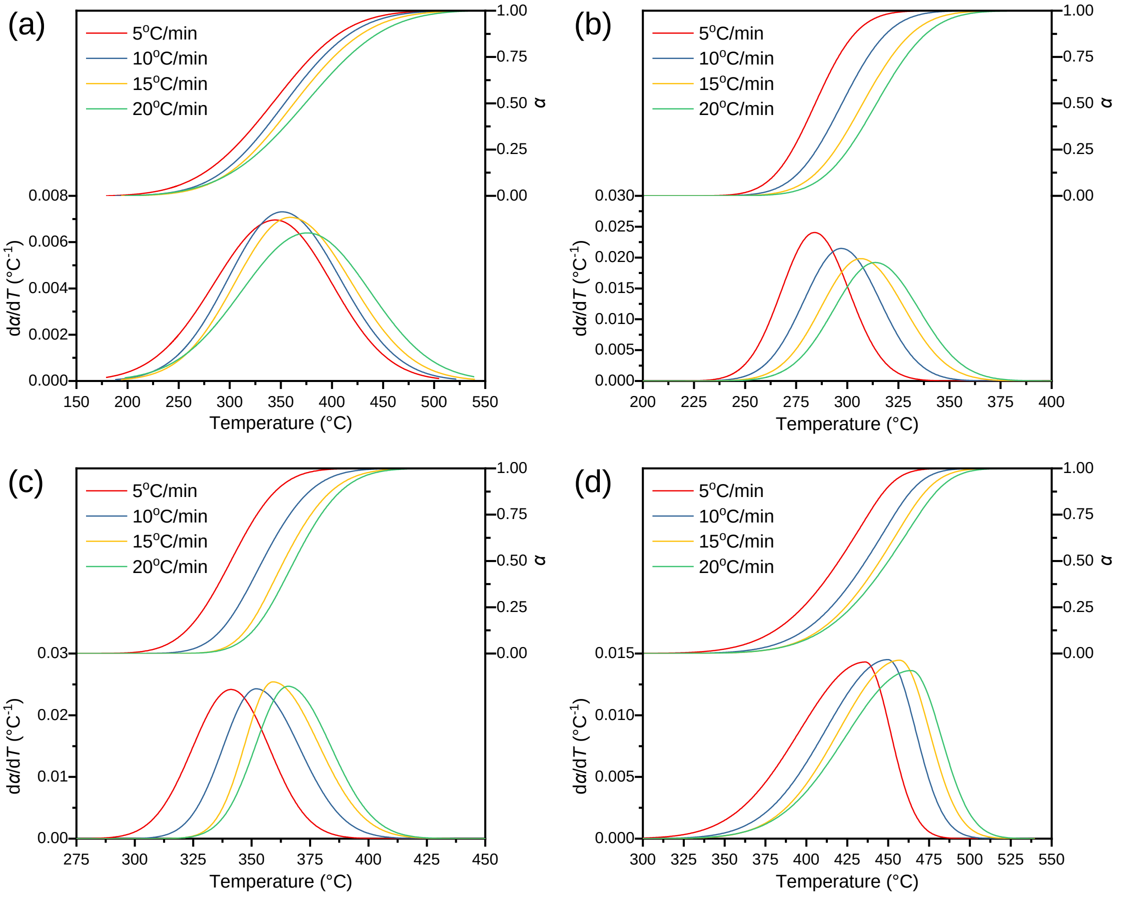

Figure S4 Normalized conversion rate curve of the deconvolution peaks of GAP-based ETPE: (a) $P_{backbone}$, (b) P_{PU1}, (c) P_{PU2}, (d) $P_{residue}$.

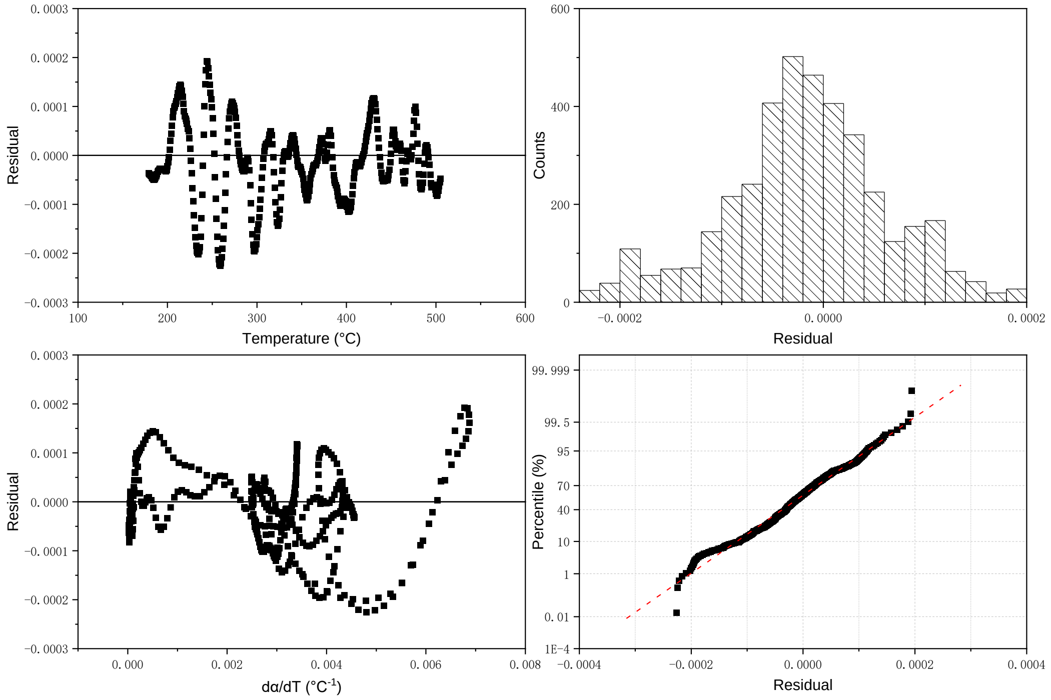

Figure S5 Residual distribution of deconvolution fitting for the dα/dT curve of ETPE with 50 wt% GAP content at a heating rate of 5 °C/min.

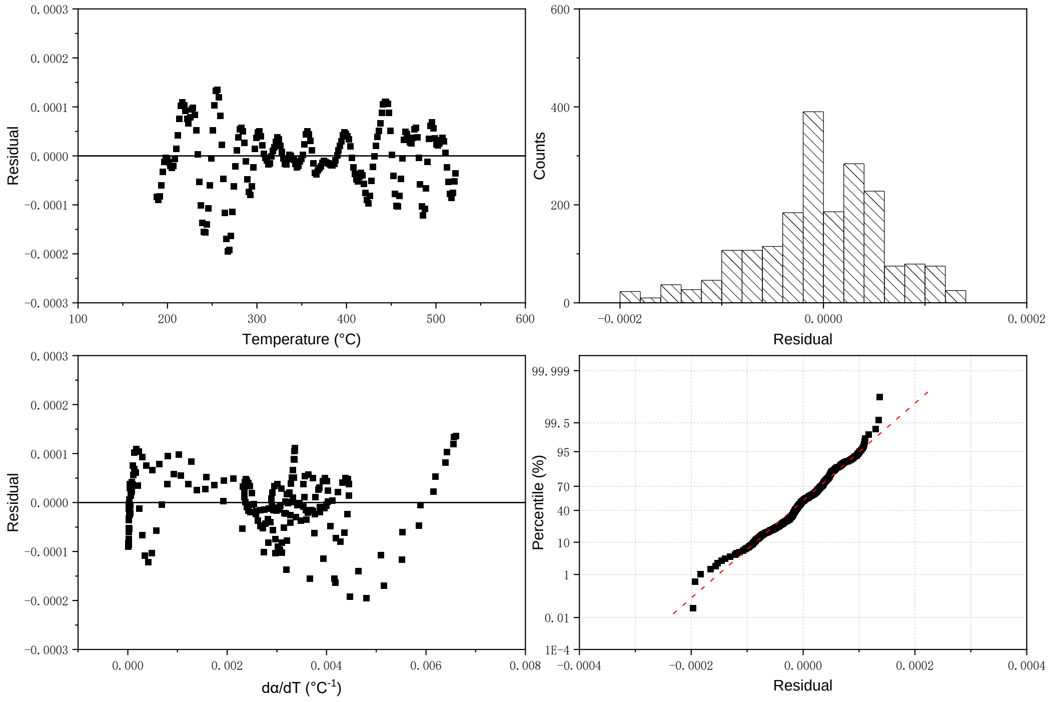

Figure S6 Residual distribution of deconvolution fitting for the dα/dT curve of ETPE with 50 wt% GAP content at a heating rate of 10 °C/min.

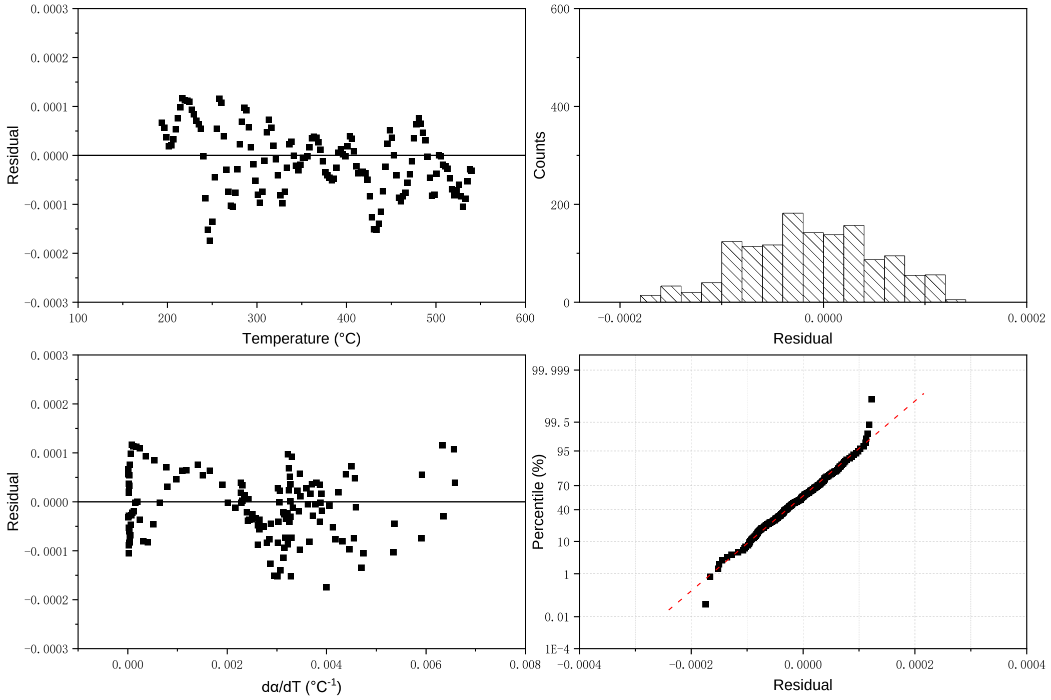

Figure S7 Residual distribution of deconvolution fitting for the dα/dT curve of ETPE with 50 wt% GAP content at a heating rate of 15 °C/min.

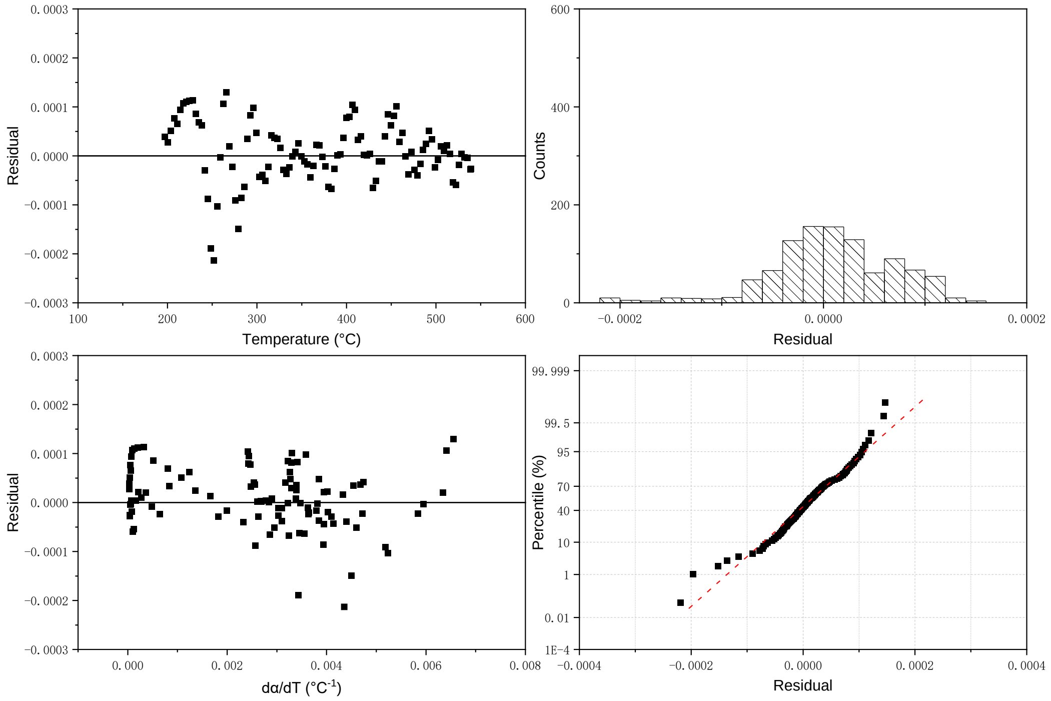

Figure S8 Residual distribution of deconvolution fitting for the dα/dT curve of ETPE with 50 wt% GAP content at a heating rate of 20 °C/min.

Table S2 $E_{\alpha,x}$ and $\ln A_{0,x}$ values of GAP-based ETPE thermal decomposition at 5 K/min derived from 17 universal kinetic mechanism functions

Model No.	P_{N3}			$P_{backbone}$			P_{PU1}			P_{PU2}			$P_{residue}$		
	E_1(kJ/mol)	$\ln A_{0,1}$	*R^2	E_2(kJ/mol)	$\ln A_{0,2}$	*R^2	E_3(kJ/mol)	$\ln A_{0,3}$	*R^2	E_4(kJ/mol)	$\ln A_{0,4}$	*R^2	E_5(kJ/mol)	$\ln A_{0,5}$	*R^2
1	162.41	36.51	0.9743	48.20	6.70	0.9913	131.99	26.92	0.9622	163.01	30.37	0.9649	151.12	24.29	0.9950
2	315.34	73.01	0.9863	92.58	16.37	0.9687	264.81	56.51	0.9902	324.56	62.94	0.9895	256.82	43.67	0.9338
3	468.26	109.51	0.9587	136.97	26.04	0.9305	390.14	88.76	0.8706	476.33	97.71	0.8689	362.51	63.04	0.8905
4	166.89	36.40	0.6093	51.51	6.20	0.6669	126.78	28.92	0.8941	156.41	32.03	0.8984	164.68	25.50	0.8499
5	228.68	50.48	0.8010	69.52	9.46	0.8482	187.85	37.53	0.7692	231.39	42.32	0.7761	207.84	32.75	0.9470
6	306.84	67.63	0.9320	92.20	12.89	0.9605	255.75	51.14	0.9165	313.97	57.46	0.9198	261.81	41.13	0.9944
7	255.87	55.46	0.8611	77.41	9.67	0.9017	211.48	41.28	0.8361	260.12	46.60	0.8415	226.58	34.67	0.9720
8	15.34	-0.21	0.9777	52.82	10.59	0.9486	37.65	6.13	0.9024	48.04	7.42	0.9149	112.32	18.52	0.9930
9	16.29	1.77	0.8165	4.39	-2.68	0.8846	6.20	-0.98	0.3862	9.72	-0.41	0.5268	42.85	4.64	0.9467
10	89.35	19.29	0.9652	26.29	2.15	0.9867	69.10	13.12	0.9431	86.36	15.12	0.9485	96.99	14.61	0.9887
11	-1.98	-2.72	0.1132	-1.09	-4.01	0.4422	-9.52	-4.61	0.7453	-9.44	-4.40	0.6769	29.31	2.03	0.8929
12	85.95	17.57	0.7032	26.00	1.17	0.7567	65.58	11.43	0.6264	82.23	13.39	0.6438	98.28	13.91	0.9575
13	60.46	11.08	0.4575	18.61	-0.85	0.5110	43.45	6.10	0.3522	55.31	7.56	0.3754	80.66	10.28	0.8764
14	88.19	18.26	0.3923	27.66	1.67	0.4471	66.72	11.85	0.3202	83.82	13.88	0.3358	105.06	15.27	0.7578
15	-69.21	-18.52	0.5532	-20.04	-7.90	0.5132	-68.37	-17.47	0.6168	-80.90	-18.56	0.6024	-14.20	-5.72	0.1107
16	-42.98	-12.98	0.2732	-12.09	-6.90	0.2422	-45.86	-13.18	0.3605	-53.45	-13.74	0.3405	5.68	-2.81	0.0169
17	-29.86	-10.29	0.1399	-8.11	-6.48	0.1168	-34.60	-11.11	0.2225	-39.72	-11.42	0.2030	15.62	-1.44	0.1077

Table S3 $E_{\alpha,x}$ and ln$A_{0,x}$ values of GAP-based ETPE thermal decomposition at 10 K/min derived from 17 universal kinetic mechanism functions

Model No.	P_{N3}			$P_{backbone}$			P_{PU1}			P_{PU2}			$P_{residual}$		
	E_1(kJ/mol)	ln$A_{0,1}$	*R^2	E_2(kJ/mol)	ln$A_{0,2}$	*R^2	E_3(kJ/mol)	ln$A_{0,3}$	*R^2	E_4(kJ/mol)	ln$A_{0,4}$	*R^2	E_5(kJ/mol)	ln$A_{0,5}$	*R^2
1	159.78	35.89	0.9705	50.34	7.64	0.9795	123.89	25.14	0.9638	152.49	28.33	0.9355	158.03	25.54	0.9983
2	313.16	71.84	0.9880	99.78	18.10	0.9780	247.88	52.21	0.9894	324.60	62.21	0.9945	273.66	46.17	0.9455
3	466.54	107.80	0.9608	149.23	28.56	0.9439	363.65	81.21	0.8677	508.03	101.63	0.8980	389.29	66.80	0.9037
4	163.92	35.67	0.5936	53.00	7.00	0.6102	118.13	26.43	0.8957	161.79	32.33	0.8691	171.10	26.62	0.8158
5	225.87	49.53	0.7910	73.02	10.57	0.8112	176.39	34.76	0.7734	219.72	39.75	0.7096	218.24	34.36	0.9287
6	304.27	66.40	0.9274	98.29	14.40	0.9430	239.77	47.10	0.9189	307.73	55.57	0.8841	277.29	43.38	0.9890
7	253.15	54.42	0.8533	81.81	10.92	0.8727	198.44	38.07	0.8396	250.36	44.28	0.7876	238.75	36.51	0.9593
8	14.27	0.26	0.9312	50.05	10.39	0.9335	35.65	6.10	0.9060	32.82	4.99	0.7733	103.17	17.52	0.9879
9	13.47	1.72	0.7123	2.25	-2.38	0.3983	6.23	-0.42	0.4191	-7.08	-2.97	0.3062	40.80	4.82	0.9669
10	86.63	18.95	0.9578	26.29	2.78	0.9650	65.06	12.50	0.9453	72.71	12.82	0.8933	99.41	15.33	0.9953
11	-4.82	-2.70	0.3773	-3.76	-3.78	0.7434	-8.47	-3.77	0.7184	-27.02	-7.03	0.9301	26.14	2.08	0.9184
12	83.09	17.21	0.6768	25.61	1.72	0.6751	61.90	10.91	0.6336	66.43	10.70	0.4920	100.21	14.54	0.9310
13	57.53	10.82	0.4215	17.37	-0.43	0.3988	41.23	6.00	0.3609	37.75	4.65	0.1945	80.94	10.70	0.8241
14	85.16	17.86	0.3685	26.94	2.15	0.3666	63.13	11.34	0.3269	65.43	10.68	0.2100	106.75	15.83	0.7025
15	-72.35	-18.29	0.5654	-25.16	-8.07	0.5767	-63.32	-15.49	0.6109	-104.68	-22.06	0.7256	-21.96	-6.28	0.1838
16	-46.10	-12.85	0.2942	-16.48	-6.95	0.3229	-42.24	-11.61	0.3530	-76.33	-17.19	0.5104	-0.51	-3.18	0.0001
17	-32.98	-10.22	0.1606	-12.14	-6.48	0.1906	-31.71	-9.75	0.2154	-62.15	-14.84	0.3766	10.22	-1.72	0.0372

Table S4 $E_{\alpha,x}$ and ln$A_{0,x}$ values of GAP-based ETPE thermal decomposition at 15 K/min derived from 17 universal kinetic mechanism functions

Model No.	P_{N3}			$P_{backbone}$			P_{PU1}			P_{PU2}			$P_{residual}$		
	E_1(kJ/mol)	ln$A_{0,1}$	*R^2	E_2(kJ/mol)	ln$A_{0,2}$	*R^2	E_3(kJ/mol)	ln$A_{0,3}$	*R^2	E_4(kJ/mol)	ln$A_{0,4}$	*R^2	E_5(kJ/mol)	ln$A_{0,5}$	*R^2
1	169.89	38.20	0.9786	49.49	7.71	0.9774	117.32	23.65	0.9625	149.05	27.69	0.9138	159.28	25.82	0.9991
2	327.15	74.66	0.9851	98.93	18.03	0.9789	236.28	49.21	0.9890	335.39	63.76	0.9948	278.87	46.87	0.9524
3	484.41	111.12	0.9561	148.37	28.34	0.9455	348.57	76.71	0.8695	550.69	108.19	0.9169	398.46	67.92	0.9115
4	175.43	38.29	0.6241	51.97	7.03	0.5990	112.74	24.84	0.8929	171.70	33.71	0.8459	171.79	26.77	0.7938
5	239.01	52.37	0.8111	71.98	10.54	0.8043	167.42	32.58	0.7696	217.53	39.16	0.6627	220.49	34.68	0.9165
6	319.38	66.40	0.9375	97.25	14.30	0.9398	228.23	44.14	0.9171	312.83	56.10	0.8580	281.57	43.92	0.9849
7	266.96	57.34	0.8693	80.77	10.86	0.8673	188.57	35.62	0.8367	250.72	44.07	0.7493	241.71	36.90	0.9505
8	13.53	0.26	0.9209	56.70	12.19	0.9605	32.79	5.70	0.9000	21.64	3.23	0.5749	98.07	16.97	0.9823
9	18.97	3.34	0.8792	1.54	-2.15	0.2324	4.61	-0.45	0.3056	-20.83	-5.09	0.7797	38.75	4.79	0.9791
10	94.43	20.91	0.9720	25.51	2.93	0.9609	60.96	11.74	0.9431	64.11	11.44	0.8458	99.02	15.45	0.9977
11	0.11	-1.16	0.0005	-4.46	-3.53	0.8009	-9.48	-3.62	0.7793	-42.06	-9.34	0.9578	23.69	2.01	0.9398
12	91.26	19.27	0.7223	24.77	1.86	0.6582	57.84	10.17	0.6243	55.88	8.97	0.3781	99.49	14.61	0.9108
13	65.05	12.79	0.4839	16.53	-0.26	0.3747	38.01	5.50	0.3460	24.83	2.55	0.0867	79.56	10.69	0.7872
14	94.03	20.07	0.4124	26.01	2.27	0.3499	58.88	10.56	0.3164	52.72	8.55	0.1322	105.74	15.83	0.6672
15	-68.77	-16.89	0.5304	-25.90	-7.76	0.5952	-62.17	-14.69	0.6292	-127.29	-25.57	0.8079	-26.35	-6.57	0.2219
16	-41.64	-11.32	0.2476	-17.25	-6.68	0.3462	-42.00	-11.07	0.3753	-97.29	-20.48	0.6323	-4.34	-3.42	0.0065
17	-28.07	-8.62	0.1186	-12.93	-6.22	0.2126	-31.91	-9.35	0.2360	-82.29	-18.01	0.5124	6.67	-1.94	0.0142

Table S5 $E_{\alpha,x}$ and ln$A_{0,x}$ values of GAP-based ETPE thermal decomposition at 20 K/min derived from 17 universal kinetic mechanism functions

Model No.	P_{N3}			$P_{backbone}$			P_{PU1}			P_{PU2}			$P_{residual}$		
	E_1(kJ/mol)	ln$A_{0,1}$	*R^2	E_2(kJ/mol)	ln$A_{0,2}$	*R^2	E_3(kJ/mol)	ln$A_{0,3}$	*R^2	E_4(kJ/mol)	ln$A_{0,4}$	*R^2	E_5(kJ/mol)	ln$A_{0,5}$	*R^2
1	156.67	35.15	0.9721	48.65	7.62	0.9908	117.02	23.56	0.9637	162.48	30.29	0.9358	153.34	24.88	0.9987
2	305.98	69.55	0.9869	93.82	17.00	0.9688	234.72	48.60	0.9892	345.58	65.53	0.9944	266.16	44.66	0.9470
3	455.30	103.95	0.9596	150.21	29.23	0.9323	344.78	75.10	0.8674	540.99	105.70	0.8980	378.99	64.45	0.9051
4	160.79	34.92	0.6004	52.00	7.09	0.6626	111.73	24.35	0.8951	172.72	33.69	0.8703	166.03	25.84	0.8108
5	239.01	48.15	0.7954	71.98	10.23	0.8460	166.87	32.33	0.7727	217.53	42.26	0.7106	212.01	33.24	0.9261
6	297.44	64.23	0.9294	93.41	13.52	0.9598	227.03	43.62	0.9189	327.70	58.77	0.8846	269.63	41.84	0.9883
7	247.67	52.76	0.8568	78.35	10.39	0.9001	187.80	35.27	0.8392	266.66	47.02	0.7884	232.02	35.24	0.9575
8	15.23	0.92	0.9752	49.78	10.75	0.9403	33.25	5.97	0.9000	35.13	6.00	0.7710	101.81	17.89	0.9859
9	14.15	2.45	0.7589	4.09	-1.48	0.8557	5.32	-0.06	0.3718	-7.31	-2.27	0.2774	39.01	4.79	0.9723
10	94.43	18.94	0.9611	26.37	3.21	0.9857	61.17	11.89	0.9449	77.58	14.15	0.8930	96.18	15.09	0.9963
11	-3.66	-1.79	0.2868	-1.49	-2.77	0.5641	-8.64	-3.17	0.7486	-28.54	-6.50	0.9172	23.69	2.39	0.9292
12	91.26	17.25	0.6889	26.07	2.23	0.7497	58.17	10.36	0.6301	55.88	11.98	0.4941	96.93	14.30	0.9259
13	57.12	11.11	0.4372	18.54	0.26	0.4991	38.55	5.78	0.3550	40.41	5.70	0.1966	78.13	10.59	0.8144
14	84.07	17.89	0.3785	27.74	2.72	0.4391	59.34	10.77	0.3232	69.89	11.96	0.2115	103.27	15.53	0.6937
15	-69.37	-16.69	0.5641	-20.78	-6.54	0.5219	-60.70	-13.99	0.6166	-111.14	-22.13	0.7248	-22.23	-5.62	0.1904
16	-43.80	-11.51	0.2888	-12.69	-5.59	0.2533	-40.69	-10.45	0.3607	-80.97	-17.04	0.5090	-1.32	-2.69	0.0007
17	-31.01	-9.01	0.1542	-8.65	-6.22	0.1263	-30.69	-8.77	0.2227	-65.88	-14.58	0.3749	9.14	-1.30	0.0306

Table S6 The parameter fitting results of the S-B model for the deconvolution peaks corresponding to the P_{N3}, P_{PU1} and P_{PU2} stages.

Decomposition Stages	Function Parameter	β=5K	β=10K	β=15K	β=20K	Average
P_{N3}	*c*	5.082	5.020	5.047	4.980	5.032
	m	0.27	0.25	0.28	0.26	0.26
	n	1.26	1.25	1.19	1.26	1.24
	R^2	0.9994	0.9996	0.9995	0.9989	0.9993
P_{PU1}	*c*	2.620	2.648	2.451	2.389	2.527
	m	0.40	0.36	0.34	0.32	0.35
	n	1.24	1.26	1.30	1.29	1.27
	R^2	0.9982	0.9981	0.9977	0.9980	0.9980
P_{PU2}	*c*	2.126	2.392	2.540	2.533	2.398
	m	0.30	0.34	0.37	0.37	0.35
	n	1.28	1.38	1.43	1.35	1.36
	R^2	0.9986	0.9967	0.9947	0.9970	0.9967
